# The Mechanism Research of Qishen Yiqi Formula by Module-Network Analysis

**DOI:** 10.1155/2015/497314

**Published:** 2015-08-24

**Authors:** Shichao Zheng, Yanling Zhang, Yanjiang Qiao

**Affiliations:** Key Laboratory of TCM-Information Engineer of State Administration of TCM, School of Chinese Pharmacy, Beijing University of Chinese Medicine, Beijing 100102, China

## Abstract

Qishen Yiqi formula (QSYQ) has the effect of tonifying Qi and promoting blood circulation, which is widely used to treat the cardiovascular diseases with Qi deficiency and blood stasis syndrome. However, the mechanism of QSYQ to tonify Qi and promote blood circulation is rarely reported at molecular or systems level. This study aimed to elucidate the mechanism of QSYQ based on the protein interaction network (PIN) analysis. The targets' information of the active components was obtained from ChEMBL and STITCH databases and was further used to search against protein-protein interactions by String database. Next, the PINs of QSYQ were constructed by Cytoscape and were analyzed by gene ontology enrichment analysis based on Markov Cluster algorithm. Finally, based on the topological parameters, the properties of scale-free, small world, and modularity of the QSYQ's PINs were analyzed. And based on function modules, the mechanism of QSYQ was elucidated. The results indicated that Qi-tonifying efficacy of QSYQ may be partly attributed to the regulation of amino acid metabolism, carbohydrate metabolism, lipid metabolism, and cAMP metabolism, while QSYQ improves the blood stasis through the regulation of blood coagulation and cardiac muscle contraction. Meanwhile, the “synergy” of formula compatibility was also illuminated.

## 1. Introduction

Qishen Yiqi formula (QSYQ), consisting of* Radix Salvia miltiorrhiza*,* Panax notoginseng*,* Dalbergia odorifera,* and* Astragalus membranaceus*, has the effect of tonifying Qi, promoting blood circulation and relieving pain, and hence it has been widely used to treat the cardiovascular diseases with Qi deficiency and blood stasis syndrome [1, 2]. Pharmacological researches have shown that the mechanism of QSYQ is related to improve myocardial function, inhibit platelet aggregation, prevent enlargement of end-diastolic diameter, and slow down ventricular remodeling [3–7]. However, the mechanism of QSYQ was mostly elucidated macroscopically by pharmacology indexes of animal experiments or clinical trials. For example, Tong et al. [6] had reported that the myocardial protection function of QSYQ may relate to the reduction of myocardial cell apoptosis in adriamycin-induced cardiomyopathy animal model. The research of Cui et al. [7] had shown that, by detecting clinical indexes which included the right ventricular end-diastolic volume (RVEDV), end-systolic volume (RVESV), stroke volume (SV), and right ventricular ejection fraction (RVEF), QSYQ could significantly improve the right heart function on patients undergoing valve replacement. The findings of these studies explain the action mechanism of QSYQ to some extent, but the further study of QSYQ is still to be done. Up to now, the mechanism of QSYQ to tonify Qi and promote blood circulation is rarely reported at molecular or systems level. In this study, the mechanism of QSYQ was illuminated by the network analysis approach which has the advantage of evaluating TCM's pharmacological effect as a whole unity at molecular level [8, 9].

Proteins are vital macromolecules, at both cellular and systematic levels, but they rarely act alone. And protein-protein interactions (PPIs) are major bearers of the biological process. So, protein interaction network (PIN) could provide the basis of understanding cellular organization and processes. The GO [10] project is a collaborative effort to construct ontologies which facilitate biologically meaningful annotation of gene products. It provides a collection of well-defined biological terms, spanning biological processes, molecular functions, and cellular components. GO enrichment is a common statistical method used to identify shared associations between proteins and annotations to GO. Module-network and GO analysis may provide an efficient way to illustrate the molecular mechanism of QSYQ.

In this study, a network analysis approach based on functional modules is applied to systematically illuminate the mechanism of QSYQ. The PINs of QSYQ were constructed by Cytoscape, while properties of scale-free, small word, and modularity were analyzed based on topological parameters. Then, the functional modules were identified by gene ontology (GO) enrichment analysis based on Markov Cluster (MCL) algorithm. This study aimed to provide an efficient way to elucidate the mechanism of QSYQ based on functional modules at the molecular level.

## 2. Materials and Methods

### 2.1. Targets Mining of Main Active Components of QSYQ

The main active components of QSYQ were used to study the mechanism of QSYQ. By literature retrieval from PubMed and CNKI database, the main active components of QSYQ were obtained based on the principles that components are the main efficacy components, have rich content, and can be absorbed into the blood. The information of the main active components of QSYQ is shown in Table 1.

The targets' information of main active components of QSYQ was obtained from two parts: pharmacophore virtual screening and the component-protein interaction database including ChEMBL (https://www.ebi.ac.uk/chembl/#) [11] and STITCH 3.1 (http://stitch.embl.de/) [12]. The 27 pharmacophore models which were applied to virtual screen were constructed by our laboratory team [13, 14]. ChEMBL is a manually curated chemical database which contains compound bioactivity data against drug targets. STITCH is a database in which every interaction has a confidence score, and the interactions with a confidence score > 0.7 were selected.

### 2.2. Network Construction of Single Herb and Formula

The PPIs information of targets was obtained from the online updated database of String 9.1 (http://string-db.org/) which has a confidence score for every protein interaction [15]. PPIs with a confidence score > 0.7 were applied to construct PIN using Cytoscape which is one of the most popular open-source software tools for the visual exploration of biomedical networks composed of protein, gene, and other types of interactions [16]. Every single herb network is formed only by PPIs involving proteins of this herb, and the formula network is formed only by PPIs involving proteins of this formula.

### 2.3. Network Analysis

The analysis of topological properties based on topological parameters has become very popular for gaining insight into the organization and structure of the resultant large complex networks [17–19]. Therefore, the topological parameters such as degree distribution, average shortest path, and clustering coefficient were analyzed by Network Analyzer [20] in Cytoscape. Properties of scale-free, small word, and modularity of the QSYQ's PIN were also investigated.

Functional modules of the network were explored by the MCL [21] which simulates a flow on the graph by calculating successive powers of the associated adjacency matrix and the value of the inflation parameter strongly influences the number of clusters. Compared to the other algorithms, for example, RNSC [22], MCODE [23], and SPC [24], the MCL is superior with highlighting the robustness to graph alterations [25]. Based on the identified modules, GO enrichment analysis was utilized to predict possible biological roles of the modules by evaluating the involved biological processes, using the BinGO [26] plugin for Cytoscape.

## 3. Results and Discussion

### 3.1. The Analysis of the Main Active Components of QSYQ

The main active components of QSYQ are all related to the effect of tonifying Qi or promoting blood stasis.

Tanshinone IIA, cryptotanshinone, salvianolic acid A, salvianolic acid B, tanshinol, and protocatechuic aldehyde are from* Salvia miltiorrhiza* which is a classical traditional Chinese medicine (TCM) which can promote blood circulation and remove blood stasis with 1000 years of clinical application [27]. It has been demonstrated that* Salvia miltiorrhiza* can reduce the area of cerebral infarct of ischemia-reperfusion injury rats which results from blood stasis [28]. The chemical components of* Salvia miltiorrhiza* are divided into water-soluble and liposoluble components. Among the liposoluble components, tanshinone IIA [29] has been reported to improve blood stasis syndrome of patients with coronary heart diseases by inhibiting the circulating inflammatory markers (including IL-6, TNF *α*, VCAM-1, CD40, sCD40L, MCP-1, and MMP9). Cryptotanshinone [30] has good pharmacological effects on atherosclerosis, while atherosclerosis is one of the diseases resulting from blood stasis. Salvianolic acids, as the main effective components of water-soluble components including salvianolic acid A, salvianolic acid B, tanshinol, and protocatechuic aldehyde, can inhibit thrombosis, thromboxane B2 formation, and platelet aggregation [31]. This indicated that the main active components from* Salvia miltiorrhiza* are all associated with blood stasis.

Dencichine, ginsenoside Rb1, ginsenoside Rg1, and notoginsenoside R1 are from* Panax notoginseng*, which is a highly-valued herb and is able to modulate vascular tone such as the activation of blood circulation, removal of blood stasis, and inhibition of platelet aggregation [32]. The main active components of* Panax notoginseng* include two types of bioactive molecules: one has been reported to have good hemostatic and antithrombotic effects, such as dencichine [33]. In addition, saponins, as the main blood-activating components, which include ginsenoside Rb1, ginsenoside Rg1, and notoginsenoside R1, have showed significant effectiveness on treating cardiovascular diseases [34, 35].

Butein, formononetin, isoliquiritigenin, and nerolidol are from* Dalbergia odorifera*.* Dalbergia odorifera*, as blood-activating and stasis-removing TCM, is widely used for promoting blood circulation, relieving pain, and removing blood stasis, which has the effects on antithrombosis, antiplatelet aggregation, antioxidant, antitumor, and anti-inflammation [36]. Volatile oil and flavonoid compounds are two main chemical components of* Dalbergia odorifera*. According to Guo et al. [37], the ethyl acetate part of* Dalbergia odorifera* can significantly shorten the bleeding time and clotting time of mice, and it indicated that volatile oil is the material basis of blood-activation in* Dalbergia odorifera*, while Nerolidol, as a main active component, accounts for 45.23~69.13% of the volatile oil. Butein, formononetin, and isoliquiritigenin, as flavonoid components, show antioxidant activity, antiplatelet aggregation, anti-inflammatory properties, and the capacity for treating cardiovascular diseases [38–40].

Calycosin, astragaloside Ι, formononetin, and astragaloside IV are from* Astragalus membranaceus* which is a popular Qi-tonifying herb with multiple biological functions, such as antioxidative, antihypertensive, antiaging, and immunomodulatory activities [41]. The main bioactive components including isoflavonoids and triterpene saponins are associated with effects on human health [42]. Isoflavonoids, which are considered “marker components” for the quality control of* Astragalus membranaceus* including calycosin and formononetin, show strong antioxidant activity, immunoregulation, anti-inflammatory properties, and the capacity for treating cardiovascular diseases [43]. Astragaloside, including astragaloside Ι and astragaloside IV, is the main effective component of astragalus polysaccharides and exerts significant effects on myocardial protection and immunity enhancement [44, 45].

### 3.2. Targets Information of Active Components of QSYQ

75 targets were obtained from pharmacophore virtual screening. 174 and 65 targets were, respectively, extracted from the ChEMBL and STITCH 3.1. The targets' number of each active component is listed in Table 2, and the additional targets' information is shown in Table S1 in Supplementary Material available online at http://dx.doi.org/10.1155/2015/497314.

### 3.3. Construction of Network

PPIs information of the targets from String 9.1 with their confidence score > 0.7 was imported in Cytoscape 2.8.3, and then union calculation was carried out, followed by the removal of duplicated edges of PPIs using Advanced Network Merge [20] of Plugins. The structural information of constructed networks was listed in Table 3.

### 3.4. Network Analysis

#### 3.4.1. Topological Analysis

All the topological parameters of QSYQ were calculated and they are shown in Table 4.

**Figure 1:**
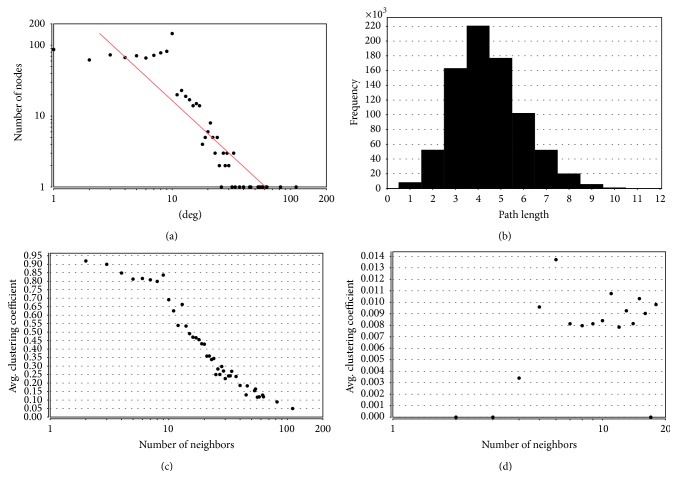
Topological properties of network. (a) The degree distribution of QSYQ network; (b) shortest path length distribution of QSYQ network; (c) average clustering coefficient of QSYQ network; (d) average clustering coefficient of random network.

Biological networks have been proposed to have scale-free topology whose degree distribution follows a power law distribution *P*(*k*) ~ *k* ^−*γ*^ (*γ* < 3) [61]. As shown in Figure 1(a), the degree distribution of the PIN of QSYQ followed the power law distribution and the equation is *y* = 582.55*x* ^−1.547^. So, the PIN of QSYQ was a scale-free network.

Small world networks have a property that mean path length is short [62]. The shortest path length between any two proteins was calculated, and it turned out to be 4.455. As shown in Figure 1(b), network path length was mostly concentrated in 3–5 steps, which meant that most proteins were closely linked and the PIN of QSYQ was a small world network.

In graph theory, a clustering coefficient is a measure of the degree to which nodes in a graph tend to cluster together. As shown in Figures 1(c) and 1(d), compared with random network whose numbers of nodes and edges are the same as PIN of QSYQ, the clustering coefficient of PIN was higher. It meant the PIN of QSYQ was more modular. These results suggested that the network exhibited the properties of scale-free, small word, and modularity.

#### 3.4.2. Clustering and GO Enrichment Analysis

**Figure 2:**
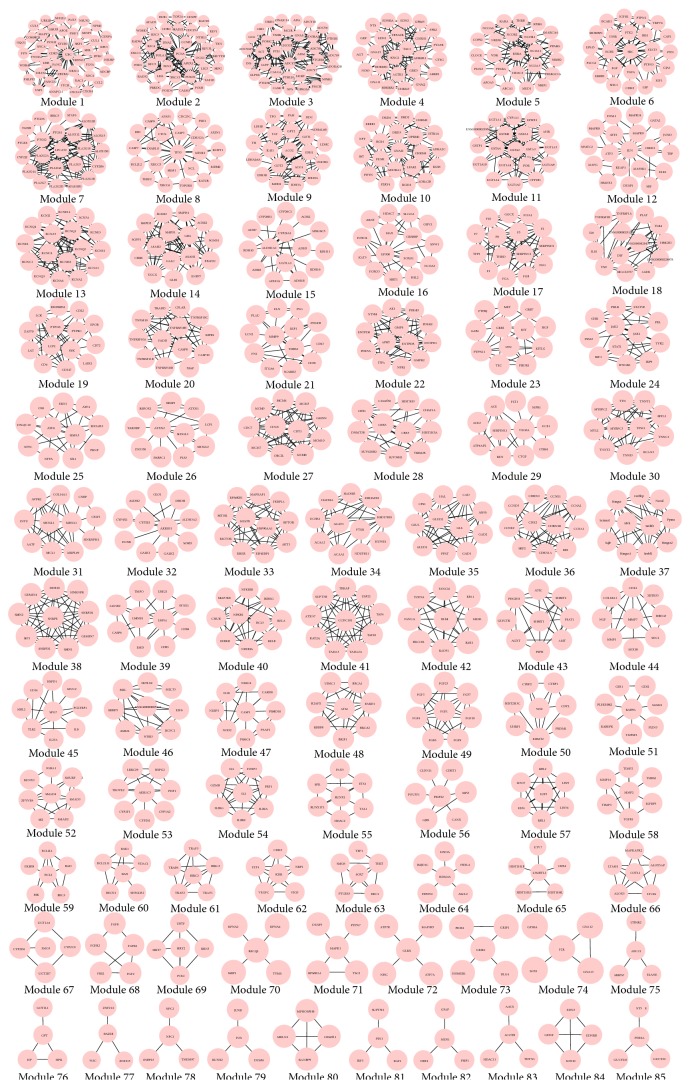
Modules in the PIN of QSYQ. With the MCL algorithm, 85 modules are extracted from the network.

With the MCL algorithm, 57, 24, 49, 29, and 85 modules were, respectively, identified from* salvia miltiorrhiza*,* Panax notoginseng*,* Dalbergia odorifera*,* Astragalus membranaceus,* and QSYQ. The modules of QSYQ are shown in Figure 2, and the others are shown in Figures S1–S4.

The results of functional enrichment analysis of QSYQ using BinGO are shown in Table 5, and they show that QSYQ played a pharmacodynamics with the biological processes, such as DNA metabolic process, regulation of cAMP metabolic process, lipid metabolic process, and the regulation of blood coagulation. The results of functional enrichment analysis of* salvia miltiorrhiza*,* Panax notoginseng*,* Dalbergia odorifera*, and* Astragalus membranaceus* are shown in Tables S2–S5.

*(1) Modules Related to Qi Deficiency*. In TCM, Qi refers to the energy which flows within our body, to support a variety of biological functions such as movement, digesting food, and fight against diseases [43]. Qi deficiency is reflected in the lack of energy. Therefore, the regulation of energy metabolism would improve the Qi deficiency. As shown in Table 5, QSYQ participated in the amino acid metabolic process, carbohydrate metabolic process, lipid metabolic process, and cAMP metabolic process which are related to the energy metabolism and also have been demonstrated to play critical roles in cardiovascular diseases [63–66]. Among them, amino acid metabolism, carbohydrate metabolism, and lipid metabolism are the main energy source of the body.

Amino acid metabolism (module 35) contained proteins such as GLUD2, GLUD1, and GLS. Glutamate dehydrogenase (GLUD) is an enzyme central to the glutamate and energy metabolism of the cell [67]. GLUD activity is raised in order to increase the amount of *α*-ketoglutarate produced, which can be used to provide energy by being used in the citric acid cycle to ultimately produce ATP. GLUD2 and GLUD1 are the GLUD's isozymes that differ in amino acid sequence but catalyze the same chemical reaction. Glutaminase (GLS) is a multifunctional enzyme involved in energy metabolism [68]. And GLS is the GLS2's isozyme, which regulates cellular energy metabolism by increasing production of glutamate and alpha-ketoglutarate and in turn results in enhanced mitochondrial respiration and ATP generation [69]. This shows that proteins in amino acid metabolism are all involved in the energy metabolism, and QSYQ can improve the Qi deficiency by regulating the amino acid metabolism.

Carbohydrate metabolism is the basis of the body to produce energy. Carbohydrate metabolism (module 32) contained proteins such as GALK1, SORD, and DCXR. Galactokinase 1 (GALK1) is an enzyme (phosphotransferase) that facilitates the phosphorylation of *α*-D-galactose to galactose 1-phosphate at the expense of one molecule of ATP. Sorbitol dehydrogenase (SORD) is an enzyme in carbohydrate metabolism converting sorbitol, the sugar alcohol form of glucose, into fructose [70]. Dicarbonyl/L-xylulose reductase (DCXR) is involved in carbohydrate metabolism and glucose metabolism which is a highly conserved and phylogenetically widespread enzyme converting L-xylulose into xylitol [71]. This shows that proteins in carbohydrate metabolism make contribution to provide energy for the body by participating in carbohydrate metabolism.

Lipid metabolism (module 83) contained proteins such as ACOT8, AACS. Acyl-coenzyme A thioesterase 8 (ACOT8) is a peroxisomal thioesterase involved more in the oxidation of fatty acids which are in order to generate acetyl-CoA, the entry molecule for the citric acid cycle, the main energy supply of animals [72]. Acetoacetyl-CoA synthetase (AACS) can directly activate ketone bodies for the synthesis of physiologically important lipidic substances such as cholesterol and fatty acid [73]. So, AACS can provide basic substances for energy metabolism. This shows that proteins in lipid metabolism are all related to energy metabolism, and QSYQ can improve the Qi deficiency by regulating the lipid metabolism.

cAMP metabolism (module 3) contained proteins such as GCG, ADCY7, and ADCYAP1. Glucagon (GCG) is a peptide hormone of cAMP metabolic process, which generally elevates the concentration of glucose in the blood by promoting gluconeogenesis and glycogenolysis [74]. Adenylate cyclase type 7 (ADCY7) is a membrane-bound adenylate cyclase that catalyses the formation of cyclic AMP from ATP [75]. ADCYAP1 is also known as pituitary adenylate cyclase-activating polypeptide (PACAP), which stimulates adenylate cyclase and subsequently increases the cAMP level and plays crucial roles in energy metabolism, including lipid metabolism [76].

This indicated that the QSYQ reinforced Qi efficacy by the regulation of the cAMP metabolism, amino acid metabolism, carbohydrate metabolism, and lipid metabolism. And Qi deficiency may be associate with the modules including amino acid metabolism, carbohydrate metabolism, lipid metabolism, and the cAMP metabolism.

*(2) Modules Related to Blood Stasis*. Blood stasis is caused by disturbance of blood circulation and is reflected in microcirculation relating to vessel and cell function, such as blood viscosity and blood cell adhesion [77]. As shown in Table 5, QSYQ took part in the regulation of blood coagulation and cardiac muscle contraction which can promote blood circulation.

The regulation of blood coagulation (module 17) contained proteins such as GGCX, F2, and SERPIND1. Gamma-glutamyl carboxylase (GGCX) catalyzes the posttranslational modification of vitamin K-dependent proteins which are involved in coagulation [78]. F2 is also known as thrombin (IIa) acts as a serine protease that converts soluble fibrinogen into insoluble strands of fibrin and activation of thrombin is crucial in physiological and pathological coagulation [79]. SERPIND1, known as heparin cofactor II, is a coagulation factor which rapidly inhibits thrombin in the presence of dermatan sulfate or heparin. SERPIND1 deficiency can lead to increased thrombin generation and a hypercoagulable state [80]. This shows that proteins in this module are all involved in the blood coagulation, and QSYQ can improve the blood stasis by the regulation of blood coagulation.

The cardiac muscle contraction (module 30) contained proteins such as MYL2, TNNC1, and TNNI3. MYL2 is also known as myosin regulatory light chain 2, ventricular/cardiac muscle isoform (MLC-2v) which plays a key role in the regulation of cardiac muscle contraction, through its interactions with myosin [81]. TNNC1 is also known as troponin C which is a protein that resides in the troponin complex on actin thin filaments of striated muscle (cardiac) and is responsible for binding calcium to activate muscle contraction [82]. Troponin I (TNNI3) has been shown to interact with TNNC1 [83] and has been reported to have a special role in the control of cardiac contractility [84]. This shows that proteins in this module are all participated in the cardiac muscle contraction. The mechanism of QSYQ has been reported to be related to improve myocardial function [7]. So, QSYQ can promote blood circulation and hence can improve the blood stasis by regulating the cardiac muscle contraction.

This indicated that QSYQ improved the blood stasis through the regulation of blood coagulation and cardiac muscle contraction. And blood stasis may be associated with the modules including the regulation of blood coagulation and cardiac muscle contraction.

#### 3.4.3. The Synergetic Effects of QSYQ

**Figure 3:**
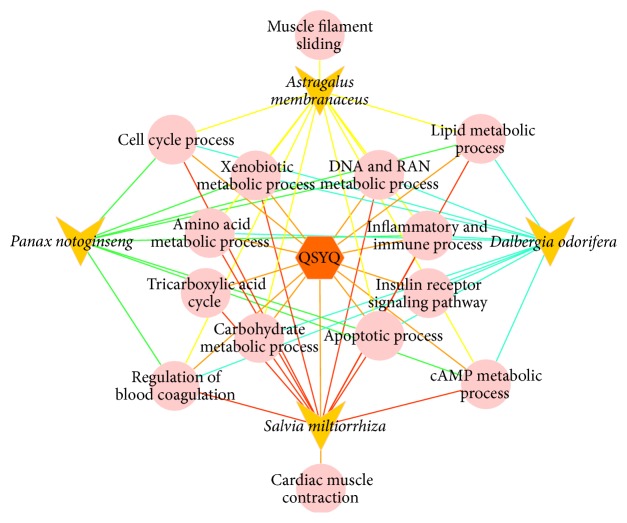
The schematic diagram of biological processes QSYQ and its herbs involved in. The hexagon represents formula. The triangle represents the herbs of QSYQ. The circle represents the biological processes.

Synergetic effects occur when the efficacy of herbs are combined. The scientific interpretation of these properties is a benefit to the explanation of the compatibility rule and it is further beneficial to the action mechanism of formulae. Synergy refers to the efficacy of combinations of herbs that is greater than the summed responses of each individual herb. As shown in Figure 3,* Salvia miltiorrhiza*,* Panax notoginseng*,* Dalbergia odorifera*, and* Astragalus membranaceus* all participate in the energy metabolism process, including cAMP metabolic process, carbohydrate metabolic process, and lipid metabolic process, and they hence have the synergetic effect on enhancing the Qi efficacy of QSYQ. The regulation of blood coagulation is involved by four herbs which reinforce the efficacy of promoting the blood circulation of QSYQ. This indicated that the synergy of formula can be illustrated based on the functional modules.

## 4. Conclusion

In this paper, the PIN of QSYQ exhibited the properties of scale-free, small world, and modularity based on the analysis of topological parameters. A module-based network analysis approach was proposed to expound the mechanism of QSYQ. Qi-tonifying efficacy of QSYQ may be partly attributed to the regulation of amino acid metabolic process, carbohydrate metabolic process, lipid metabolic process, and the cAMP metabolic process, while QSYQ improves the blood stasis through the regulation of blood coagulation and cardiac muscle contraction. A systematic exploration of mechanism of QSYQ based on module-network analysis may bring out the best between research on drug molecules and TCM phenotypic information, so as to facilitate the therapy for the disease.

Further experiments are needed to confirm the conclusions. However, despite the lack of validation of wet experiments, this study provides an efficient way to understand the mechanisms of QSYQ faster and better considering the complexity of TCM analogous formulae. What is more, the scientific intension of “synergy” of TCM can be also illustrated based on the functional modules at the molecular level.

## Supplementary Material

Table S1 is about the additional targets' information of each active components from QSYQ. Tables S2–S5 are respective about the results of functional enrichment analysis of salvia miltiorrhiza, Panax notoginseng, Dalbergia odorifera,and Astragalus membranaceus. Figures S1–S4 are respective about the Modules in the PIN of salvia miltiorrhiza, panax notoginseng, dalbergia odorifera, astragalus membranaceus.

## Figures and Tables

**Table 1:** The information of the main active components of QSYQ.

Herbs	Active components	Reference
*Salvia miltiorrhiza*	Tanshinone IIA, cryptotanshinone, salvianolic acid A, salvianolic acid B, tanshinol, and protocatechuic aldehyde	[46–51]
*Panax notoginseng*	Dencichine, ginsenoside Rb1, ginsenoside Rg1, and notoginsenoside R1	[52–54]
*Dalbergia odorifera*	Butein, formononetin, isoliquiritigenin, nerolidol	[55–58]
*Astragalus membranaceus*	Calycosin, astragaloside Ι, formononetin, and astragaloside IV	[59, 60]

**Table 2:** The targets' number of each active component from QSYQ.

Active components	total
Tanshinone IIA	45
Cryptotanshinone	34
Salvianolic acid A	17
Salvianolic acid B	21
Tanshinol	8
Protocatechuic aldehyde	9
Dencichine	7
Ginsenoside Rb1	15
Ginsenoside Rg1	18
Notoginsenoside R1	11
Butein	23
Formononetin	27
Isoliquiritigenin	51
Nerolidol	3
Calycosin	8
Astragaloside Ι	7
Astragaloside IV	10

**Table 3:** The structural information of networks on herbs and formula.

Networks	Nodes	Edges
*Salvia miltiorrhiza*	604	2362
*Panax notoginseng*	264	963
*Dalbergia odorifera*	588	2379
*Astragalus membranaceus*	399	1294
QSYQ	993	4215

**Table 4:** The simple parameters of protein interaction network of QSYQ.

Parameters	PIN of QSYQ
Clustering coefficient	0.673
Network diameter (radius)	11 (1)
Network centralization	0.104
Shortest path	804676
Mean path length	4.455
Network heterogeneity	0.955

*Notes*. The network diameter is the longest distance between any pair of vertices and the radius of a graph is the minimum eccentricity of any vertex. Network centralization is a network index that measures the degree of dispersion of all node centrality scores in a network. And network heterogeneity quantifies the degree of uneven distribution of the network.

**Table 5:** GO biological process terms of the modules of QSYQ.

Modules	*P* value	GO terms
1	1.97*E* − 14	Regulation of protein metabolic process
2	1.32*E* − 32	DNA metabolic process
3	2.01*E* − 28	Regulation of cAMP metabolic process
4	4.16*E* − 24	G-protein coupled receptor signaling pathway
5	5.28*E* − 23	DNA-dependent transcription, initiation
6	3.03*E* − 25	Transmembrane receptor protein tyrosine kinase signaling pathway
7	4.74*E* − 23	Cellular lipid metabolic process
8	6.43*E* − 15	Apoptotic process
9	1.80*E* − 18	Tricarboxylic acid cycle
10	9.96*E* − 27	G-protein coupled receptor signaling pathway
11	1.32*E* − 32	Xenobiotic metabolic process
12	2.74*E* − 15	Toll-like receptor signaling pathway
13	5.96*E* − 32	Potassium ion transport
14	1.50*E* − 20	Lipid metabolic process
15	2.57*E* − 20	Xenobiotic metabolic process
16	8.66*E* − 12	Positive regulation of RNA metabolic process
17	2.78*E* − 27	Regulation of blood coagulation
18	3.33*E* − 14	Inflammatory response
19	1.94*E* − 16	Immune response-activating signal transduction
20	3.65*E* − 19	Apoptotic process
21	1.04*E* − 11	Regulation of blood coagulation
22	2.64*E* − 16	Nucleotide metabolic process
23	4.14*E* − 13	Transmembrane receptor protein tyrosine kinase signaling pathway
24	3.85*E* − 12	Interferon-gamma-mediated signaling pathway
25	5.59*E* − 07	Regulation of cellular protein metabolic process
26	4.34*E* − 06	RNA processing
27	1.19*E* − 22	Cell cycle phase transition
28	2.71*E* − 06	Regulation of RNA metabolic process
29	9.03*E* − 14	Regulation of systemic arterial blood pressure by renin-angiotensin
30	5.05*E* − 18	Cardiac muscle contraction
31	2.53*E* − 06	Regulation of RNA splicing
32	1.13*E* − 09	Carbohydrate metabolic process
33	9.21*E* − 14	Insulin receptor signaling pathway
34	3.77*E* − 08	Lipid catabolic process
35	2.50*E* − 22	Cellular amino acid catabolic process
36	1.93*E* − 17	Regulation of cell cycle
37	2.42*E* − 14	Lipid metabolic process
38	3.52*E* − 17	mRNA metabolic process
39	2.31*E* − 06	Execution phase of apoptosis
40	1.16*E* − 15	Toll-like receptor signaling pathway
41	3.70*E* − 11	RNA biosynthetic process
42	3.65*E* − 12	Cell cycle phase
43	2.80*E* − 12	Cellular amino acid metabolic process
44	6.84*E* − 05	Regulation of cell proliferation
45	1.17*E* − 07	Inflammatory response
46	4.51*E* − 09	RNA biosynthetic process
47	3.74*E* − 11	Regulation of interleukin-1 secretion
48	1.49*E* − 13	DNA repair
49	1.67*E* − 18	Insulin receptor signaling pathway
50	3.18*E* − 06	Negative regulation of RNA metabolic process
51	1.24*E* − 04	Transport
52	8.34*E* − 14	Regulation of transforming growth factor beta receptor signaling pathway
53	4.47*E* − 06	Xenobiotic metabolic process
54	1.16*E* − 08	Negative regulation of inflammatory response
55	1.37*E* − 07	Positive regulation of RNA metabolic process
56	5.56*E* − 07	Transmission of nerve impulse
57	5.79*E* − 12	Mitotic cell cycle
58	6.69*E* − 06	Negative regulation of protein metabolic process
59	2.66*E* − 12	Regulation of apoptotic signaling pathway
60	2.00*E* − 09	Regulation of apoptotic signaling pathway
61	2.47*E* − 11	NIK/NF-kappaB cascade
62	4.69*E* − 15	Vascular endothelial growth factor receptor signaling pathway
63	1.98*E* − 06	DNA metabolic process
64	2.04*E* − 04	Chromatin organization
65	1.11*E* − 05	DNA packaging
66	9.39*E* − 07	Inflammatory response
67	3.22*E* − 09	Xenobiotic metabolic process
68	8.09*E* − 12	Insulin receptor signaling pathway
69	8.52*E* − 04	DNA-dependent transcription, initiation
70	1.56*E* − 05	DNA replication
71	5.51*E* − 07	Toll-like receptor signaling pathway
72	2.16*E* − 08	Copper ion import
73	2.90*E* − 08	Transmission of nerve impulse
74	4.63*E* − 07	Regulation of blood coagulation
76	6.90*E* − 04	Alanine catabolic process
77	6.76*E* − 05	RNA biosynthetic process
78	7.60*E* − 04	Lipid metabolic process
79	2.86*E* − 05	TRIF-dependent toll-like receptor signaling pathway
81	4.18*E* − 05	Regulation of type I interferon production
83	5.21*E* − 03	Cellular lipid metabolic process
84	1.27*E* − 11	Neural crest cell migration
85	2.10*E* − 03	AMP catabolic process

*Notes*. *P* value is the probability of obtaining the observed effect under the null hypothesis; a very small *P* value indicates that the observed effect is very unlikely to have arisen purely by chance.
