# Supplementary material for: The Mechanism Research of Qishen Yiqi Formula by Module-Network Analysis
Source: Evid Based Complement Alternat Med. 2015 Aug 24;2015:497314. doi: 10.1155/2015/497314 (PMC4561322; doi:10.1155/2015/497314)
Supplement: Supplementary file 1 — Table S1 is about the additional targets' information of each active components from QSYQ. Tables S2–S5 are respective about the results of functional enrichment analysis of salvia miltiorrhiza, Panax notoginseng, Dalbergia odorifera,and Astragalus membranaceus. Figures S1–S4 are respective about the Modules in the PIN of salvia miltiorrhiza, panax notoginseng, dalbergia odorifera, astragalus membranaceus. [file 497314.f1.zip › supplementary materials/FIGURE S1. Modules in the PIN of salvia miltiorrhiza.pdf]

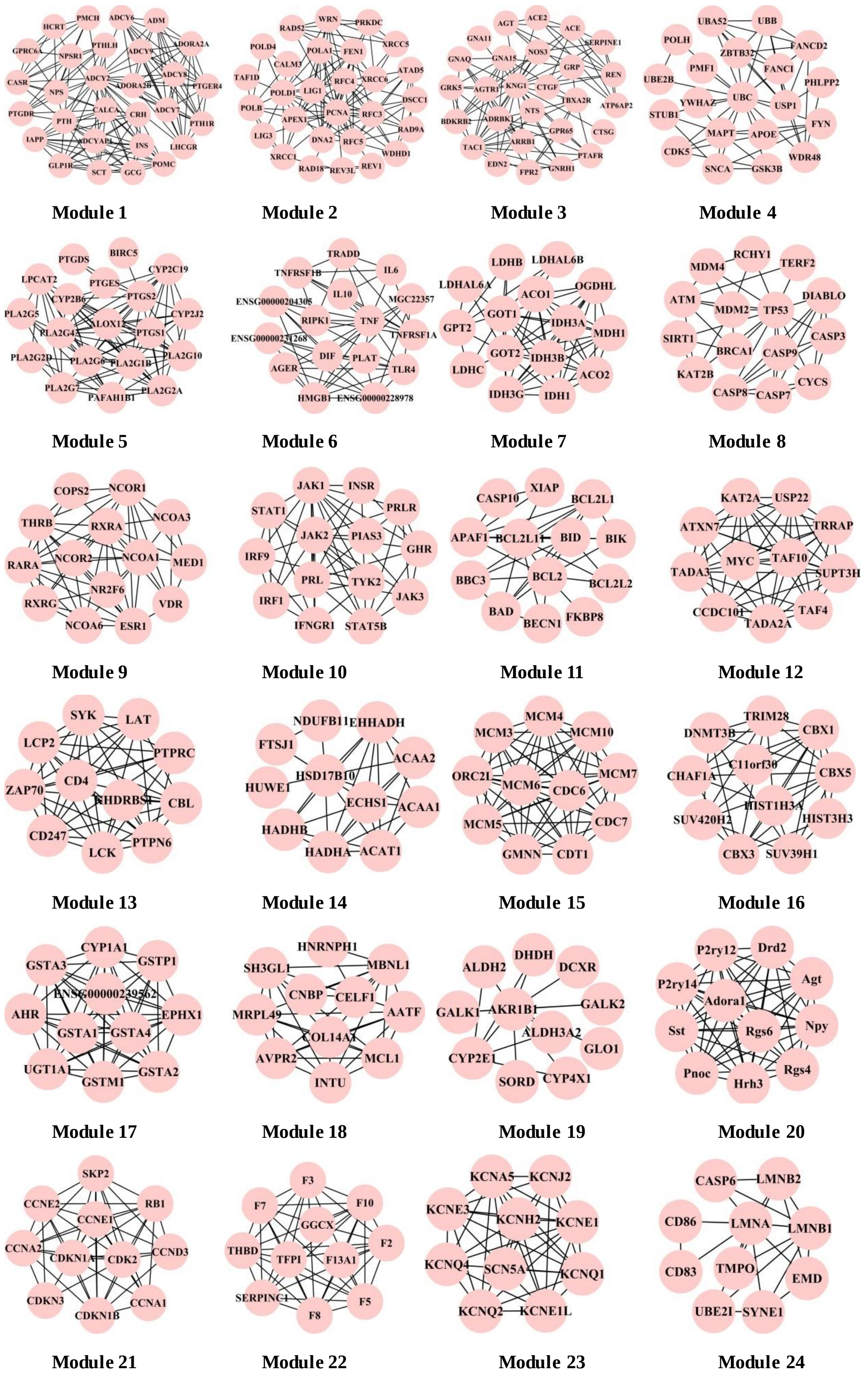
HCRT PMCH ADCY6 ADM GPRC6A NPSR1 PTHLH ADCY9 ADORA2A CASR NPS ADCY2 ADORA2B ADCY8 PTGER4 PTGDR PTH CALCA CRH ADCY7 PTH1R IAPP ADCYAP1 INS LHCGR GLP1R SCT GCG POMC
Module 1
WRN PRKDC RAD52 POLD4 POLA1 FEN1 XRCC5 CALM3 TAF1D RFC4 XRCC6 ATAD5 POLD1 LIG1 DSCC1 POLB PCNA RFC3 APEX1 RAD9A LIG3 DNA2 RFC5 XRCC1 WDHD1 RAD18 REV3L REV1
Module 2
AGT ACE2 ACE SERPINE1 GNA11 NOS3 GNAQ GNA15 GRP REN GRK5 AGTR1 KNG1 CTGF ATP6AP2 TBXA2R BDKRB2 ADRBK1 NTS TAC1 ARRB1 GPR65 CTSG EDN2 PTAFR FPR2 GNRH1
Module 3
UBA52 UBB POLH FANCD2 ZBTB32 PMF1 FANCI UBE2B PHLPP2 UBC USP1 YWHAZ STUB1 FYN MAPT APOE CDK5 WDR48 SNCA GSK3B
Module 4
PTGDS BIRC5 LPCAT2 CYP2C19 PTGES CYP2B6 PTGS2 PLA2G5 CYP2J2 ALOX12 PLA2G4A PTGS1 PLA2G2D PLA2G10 PLA2G6 PLA2G1B PLA2G7 PAFAH1B1 PLA2G2A
Module 5
TRADD TNFRSF1B IL6 IL10 ENSG00000204305 MGC22357 RIPK1 TNF ENSG00000231268 TNFRSF1A DIF PLAT AGER TLR4 HMGB1 ENSG00000228978
Module 6
LDHB LDHAL6B LDHAL6A OGDHL ACO1 GOT1 GPT2 IDH3A MDH1 GOT2 IDH3B LDHC ACO2 IDH3G IDH1
Module 7
RCHY1 MDM4 TERF2 DIABLO ATM MDM2 TP53 SIRT1 CASP3 BRCA1 CASP9 KAT2B CYCS CASP8 CASP7
Module 8
NCOR1 COPS2 NCOA3 THRB RXRA NCOR2 NCOA1 MED1 RARA NR2F6 RXRG VDR NCOA6 ESR1
Module 9
JAK1 INSR PRLR STAT1 JAK2 PIAS3 GHR IRF9 PRL TYK2 IRF1 JAK3 IFNGR1 STAT5B
Module 10
XIAP CASP10 BCL2L1 APAF1 BCL2L11 BID BIK BBC3 BCL2 BCL2L2 BAD FKBP8 BECN1
Module 11
KAT2A USP22 ATXN7 TRRAP MYC TAF10 TADA3 SUPT3H CCDC101 TAF4 TADA2A
Module 12
SYK LAT LCP2 PTPRC CD4 ZAP70 SH2D1A CBL CD247 PTPN6 LCK
Module 13
NDUFB11 EHHADH FTSJ1 ACAA2 HSD17B10 HUWE1 ECHS1 ACAA1 HADHB HADHA ACAT1
Module 14
MCM4 MCM3 MCM10 ORC2L MCM6 CDC6 MCM7 MCM5 CDC7 GMNN CDT1
Module 15
TRIM28 DNMT3B CBX1 C11orf30 CBX5 CHAF1A HIST1H3A SUV420H2 HIST3H3 CBX3 SUV39H1
Module 16
CYP1A1 GSTA3 GSTP1 ENSG00000239562 EPHX1 AHR GSTA1 GSTA4 UGT1A1 GSTA2 GSTM1
Module 17
HNRNPH1 SH3GL1 MBNL1 CNBP CELF1 MRPL49 AATF COL14A1 AVPR2 MCL1 INTU
Module 18
DHDH ALDH2 DCXR GALK2 GALK1 AKR1B1 ALDH3A2 GLO1 CYP2E1 CYP4X1 SORD
Module 19
P2ry12 Drd2 P2ry14 Agt Adora1 Npy Sst Rgs6 Pnoc Rgs4 Hrh3
Module 20
SKP2 CCNE2 RB1 CCNE1 CCNA2 CDKN1A CDK2 CCND3 CDKN3 CCNA1 CDKN1B
Module 21
F3 F7 F10 GGCX F2 THBD TFPI F13A1 SERPINC1 F5 F8
Module 22
KCNA5 KCNJ2 KCNE3 KCNH2 KCNE1 KCNQ4 SCN5A KCNQ1 KCNQ2 KCNE1L
Module 23
CASP6 LMNB2 CD86 LMNA LMNB1 CD83 TMPO EMD UBE2I SYNE1
Module 24

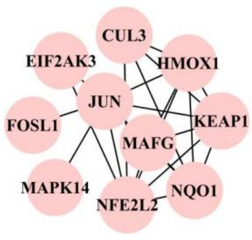

Module 25

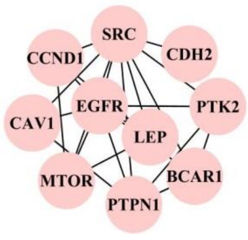

Module 26

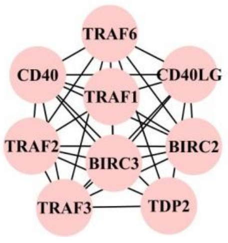

Module 27

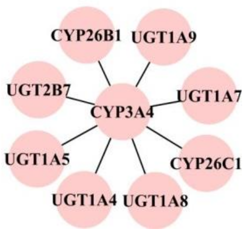

Module 28

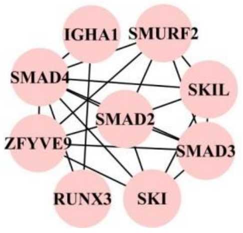

Module 29

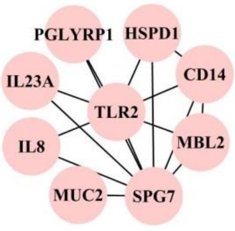

Module 30

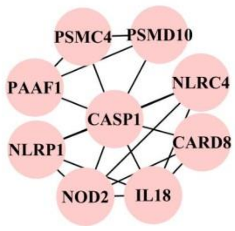

Module 31

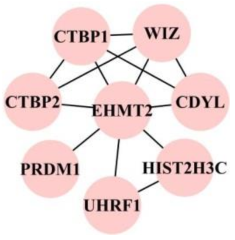

Module 32

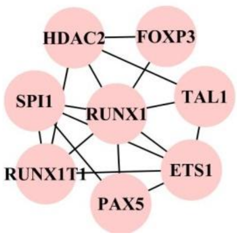

Module 33

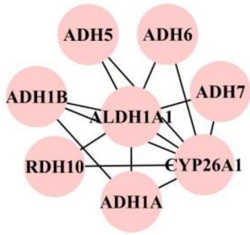

Module 34

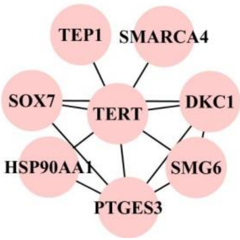

Module 35

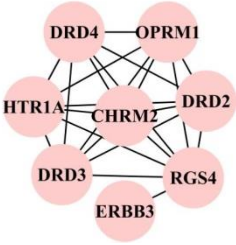

Module 36

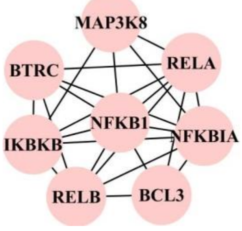

Module 37

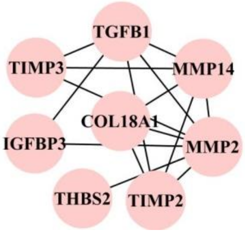

Module 38

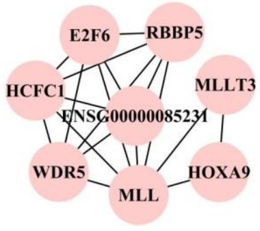

Module 39

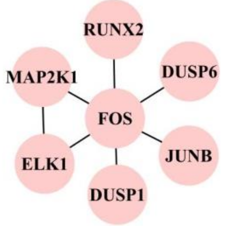

Module 40

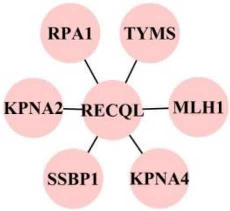

Module 41

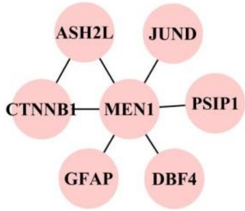

Module 42

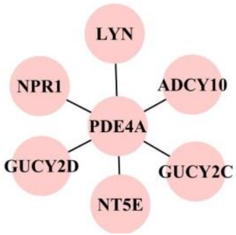

Module 43

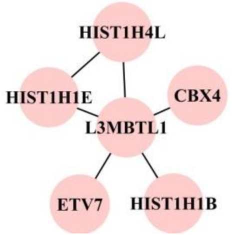

Module 44

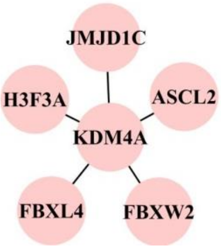

Module 45

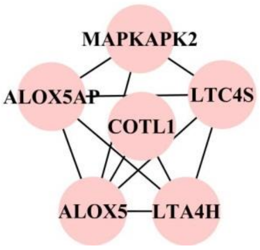

Module 46

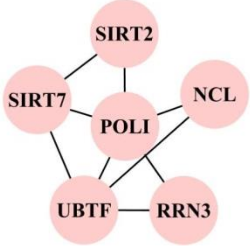

Module 47

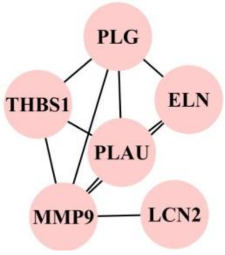

Module 48

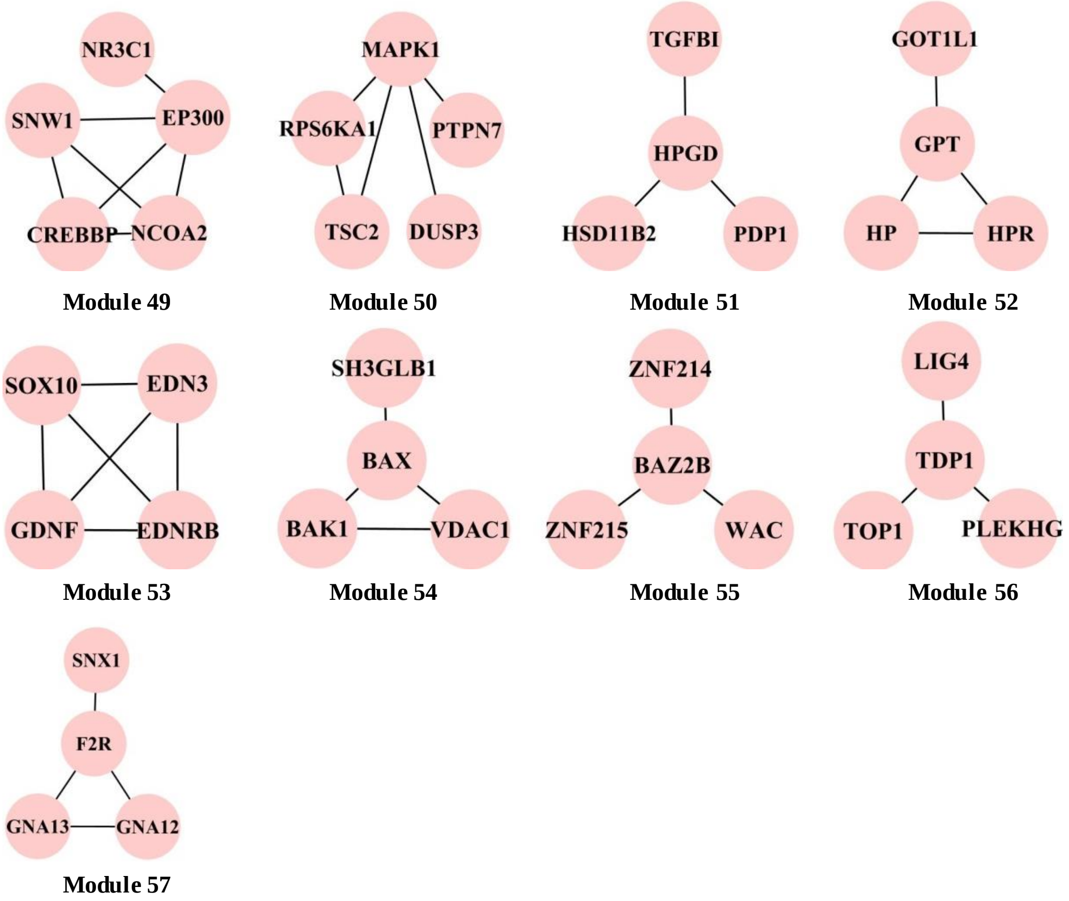

FIGURE S1: Modules in the PIN of *salvia miltiorrhiza*. With the MCL algorithm, 57 modules are extracted from the network.
