# Supplementary material for: The Mechanism Research of Qishen Yiqi Formula by Module-Network Analysis
Source: Evid Based Complement Alternat Med. 2015 Aug 24;2015:497314. doi: 10.1155/2015/497314 (PMC4561322; doi:10.1155/2015/497314)
Supplement: Supplementary file 1 — Table S1 is about the additional targets' information of each active components from QSYQ. Tables S2–S5 are respective about the results of functional enrichment analysis of salvia miltiorrhiza, Panax notoginseng, Dalbergia odorifera,and Astragalus membranaceus. Figures S1–S4 are respective about the Modules in the PIN of salvia miltiorrhiza, panax notoginseng, dalbergia odorifera, astragalus membranaceus. [file 497314.f1.zip › supplementary materials/FIGURE S3. Modules in the PIN of dalbergia odorifera..pdf]

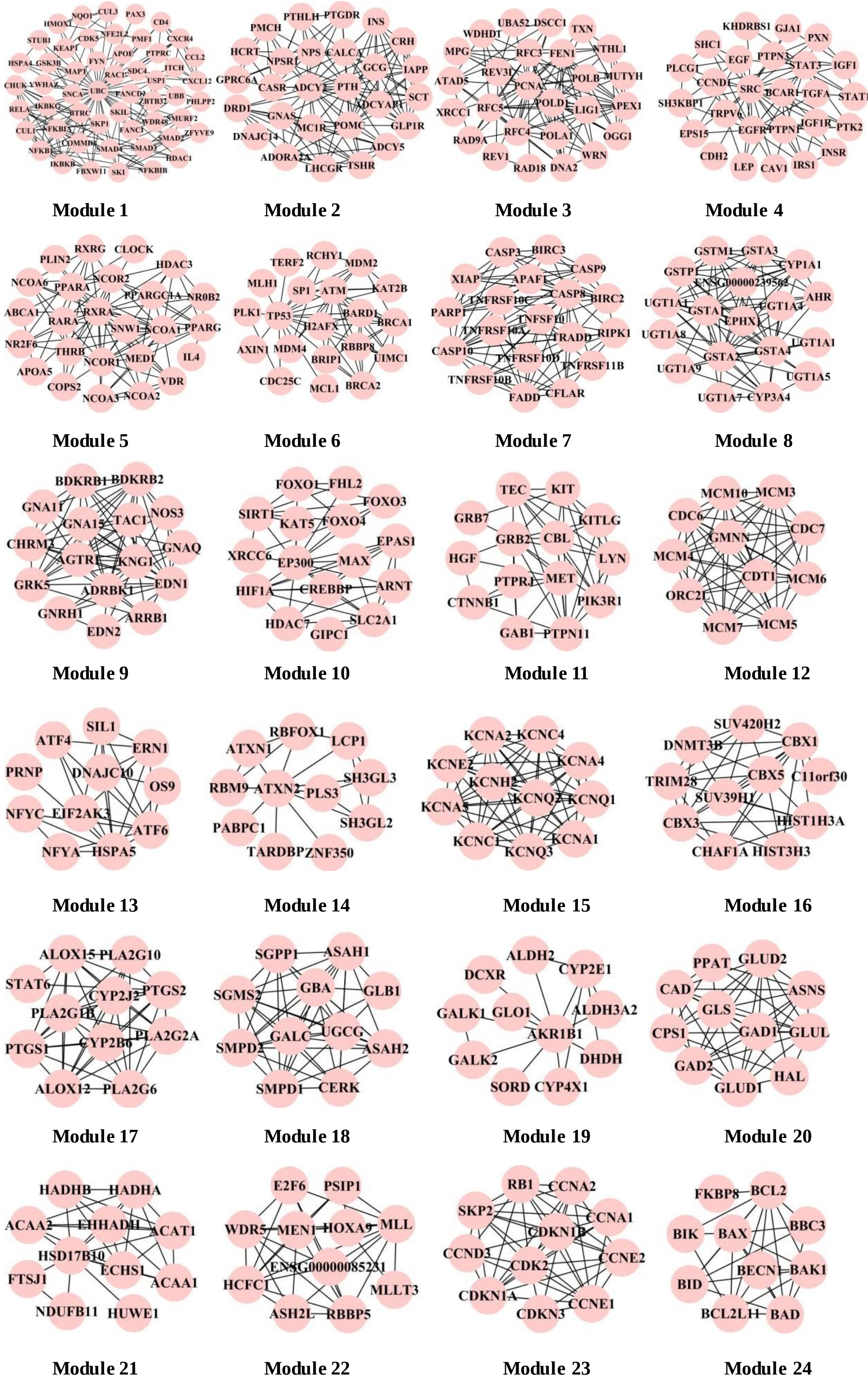

Module 1
PTHLH PTGDR INS PMCH CRH HCRT NPS CALCA NPSR1 GCG IAPP GPRC6A CASR ADCY2 PTH SCT DRD1 ADCYAP1 GNAS GLP1R MC1R POMC DNAJC14 ADCY5 ADORA2A LHCGR TSHR
Module 2
UBA52 DSCC1 TXN WDHD1 MPG RFC3 FEN1 NTHL1 REV3L POLB MUTYH ATAD5 PCNA POLD1 XRCC1 RFC5 LIG1 APEX1 RAD9A RFC4 POLA1 OGG1 REV1 RAD18 DNA2 WRN
Module 3
KHDRBS1 GJA1 PXN SHC1 EGF PTPN3 STAT3 IGF1 PLCG1 CCND1 SRC BCAR1 TGFA STAT1 SH3KBP1 TRPV6 IGF1R PTK2 EPS15 EGFR PTPN1 CDH2 LEP CAV1 IRS1 INSR
Module 4
RXRG CLOCK PLIN2 NCOR2 HDAC3 NCOA6 PPARA PPARGC1A NR0B2 ABCA1 RARA RXRA PPARG NR2F6 SNW1 NCOA1 THRB IL4 NCOR1 MED1 APOA5 VDR COPS2 NCOA3 NCOA2
Module 5
RCHY1 TERF2 MDM2 KAT2B MLH1 SP1 ATM PLK1 TP53 BARD1 H2AFX BRCA1 AXIN1 MDM4 RBBP8 UIMC1 BRIP1 CDC25C MCL1 BRCA2
Module 6
CASP3 BIRC3 CASP9 XIAP APAF1 TNFRSF10C CASP8 BIRC2 PARP1 TNFSF10 TNFRSF10A RIPK1 CASP10 TRADD TNFRSF10D TNFRSF11B TNFRSF10B FADD CFLAR
Module 7
GSTM1 GSTA3 CYP1A1 GSTP1 ENSG00000239562 AHR UGT1A1 GSTA1 UGT1A4 EPHX1 UGT1A8 GSTA2 GSTA4 UGT1A1 UGT1A9 UGT1A5 UGT1A7 CYP3A4
Module 8
BDKRB1 BDKRB2 GNA11 NOS3 GNA15 TAC1 CHRM2 GNAQ AGTR1 KNG1 GRK5 EDN1 ADRBK1 GNRH1 ARRB1 EDN2
Module 9
FOXO1 FHL2 FOXO3 SIRT1 KAT5 FOXO4 EPAS1 XRCC6 EP300 MAX ARNT HIF1A CREBBP HDAC7 SLC2A1 GIPC1
Module 10
TEC KIT GRB7 KITLG GRB2 CBL LYN HGF PTPRJ MET PIK3R1 CTNNB1 GAB1 PTPN11
Module 11
MCM10 MCM3 CDC6 CDC7 GMNN MCM4 CDT1 MCM6 ORC2L MCM7 MCM5
Module 12
SIL1 ATF4 ERN1 PRNP DNAJC10 OS9 NFYC EIF2AK3 ATF6 NFYA HSPA5
Module 13
RBFOX1 ATXN1 LCP1 SH3GL3 RBM9 ATXN2 PLS3 PABPC1 SH3GL2 TARDBP ZNF350
Module 14
KCNA2 KCNC4 KCNE2 KCNA4 KCNH2 KCNQ2 KCNQ1 KCNA5 KCNC1 KCNA1 KCNQ3
Module 15
SUV420H2 DNMT3B CBX1 CBX5 C11orf30 TRIM28 SUV39H1 CBX3 HIST1H3A CHAF1A HIST3H3
Module 16
ALOX15 PLA2G10 STAT6 CYP2J2 PTGS2 PLA2G1B PLA2G2A PTGS1 CYP2B6 ALOX12 PLA2G6
Module 17
SGPP1 ASAH1 SGMS2 GBA GLB1 SMPD2 GALC UGCG ASAH2 SMPD1 CERK
Module 18
ALDH2 DCXR CYP2E1 GALK1 GLO1 ALDH3A2 AKR1B1 GALK2 DHDH SORD CYP4X1
Module 19
PPAT GLUD2 CAD ASNS GLS CPS1 GAD1 GLUL GAD2 HAL GLUD1
Module 20
HADHB HADHA ACAA2 EHHADH ACAT1 HSD17B10 FTSJ1 ECHS1 ACAA1 NDUFB11 HUWE1
Module 21
E2F6 PSIP1 WDR5 MEN1 HOXA9 MLL ENSG00000085231 HCFC1 MLLT3 ASH2L RBBP5
Module 22
RB1 CCNA2 SKP2 CCNA1 CDKN1B CCND3 CCNE2 CDK2 CDKN1A CCNE1 CDKN3
Module 23
FKBP8 BCL2 BIK BAX BBC3 BECN1 BAK1 BID BCL2L11 BAD
Module 24

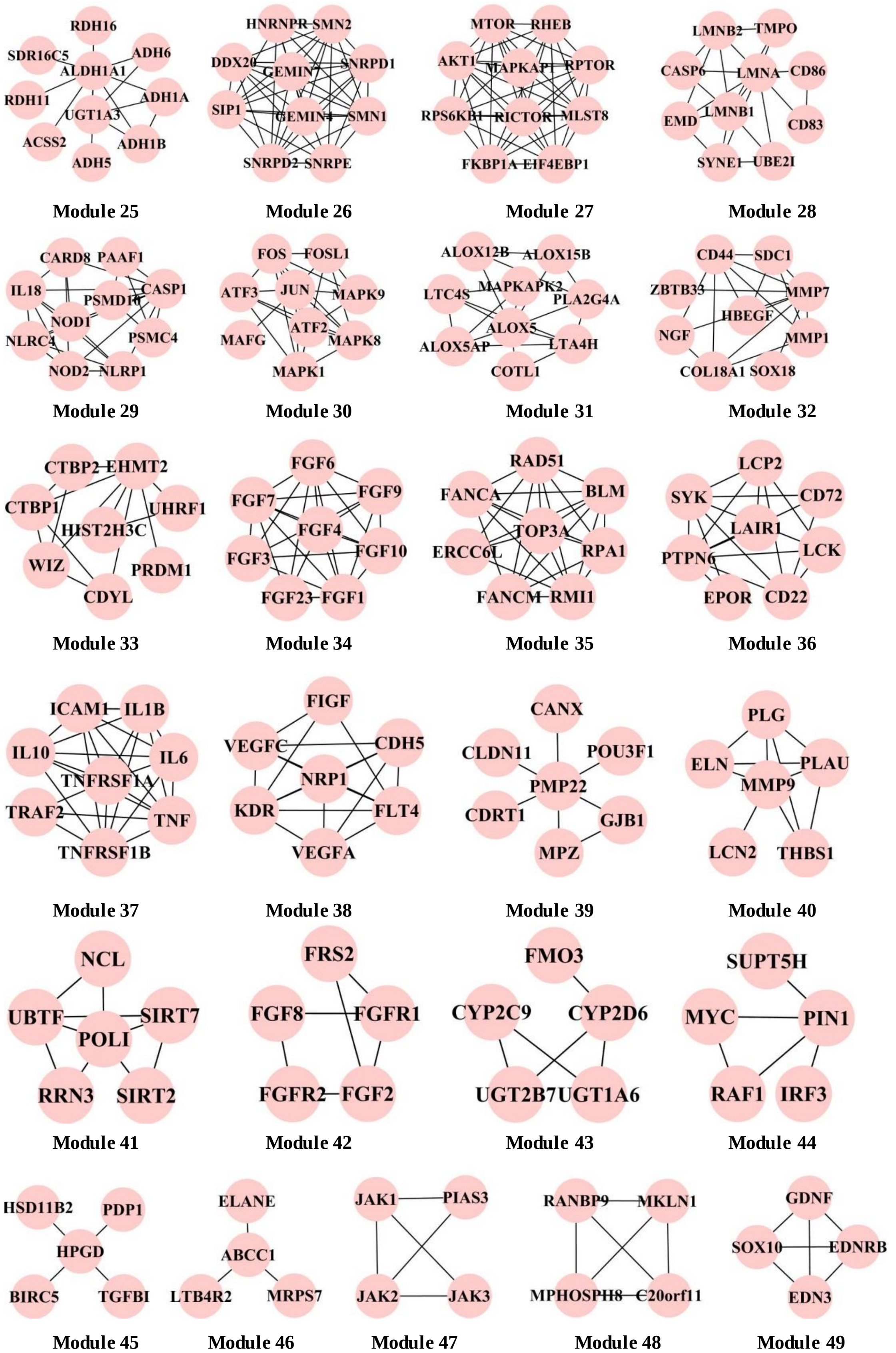

FIGURE S3: Modules in the PIN of *dalbergia odorifera*. With the MCL algorithm, 49 modules are extracted from the network.
