# Supplementary material for: The Mechanism Research of Qishen Yiqi Formula by Module-Network Analysis
Source: Evid Based Complement Alternat Med. 2015 Aug 24;2015:497314. doi: 10.1155/2015/497314 (PMC4561322; doi:10.1155/2015/497314)
Supplement: Supplementary file 1 — Table S1 is about the additional targets' information of each active components from QSYQ. Tables S2–S5 are respective about the results of functional enrichment analysis of salvia miltiorrhiza, Panax notoginseng, Dalbergia odorifera,and Astragalus membranaceus. Figures S1–S4 are respective about the Modules in the PIN of salvia miltiorrhiza, panax notoginseng, dalbergia odorifera, astragalus membranaceus. [file 497314.f1.zip › supplementary materials/FIGURE S4. Modules in the PIN of astragalus membranaceus.pdf]

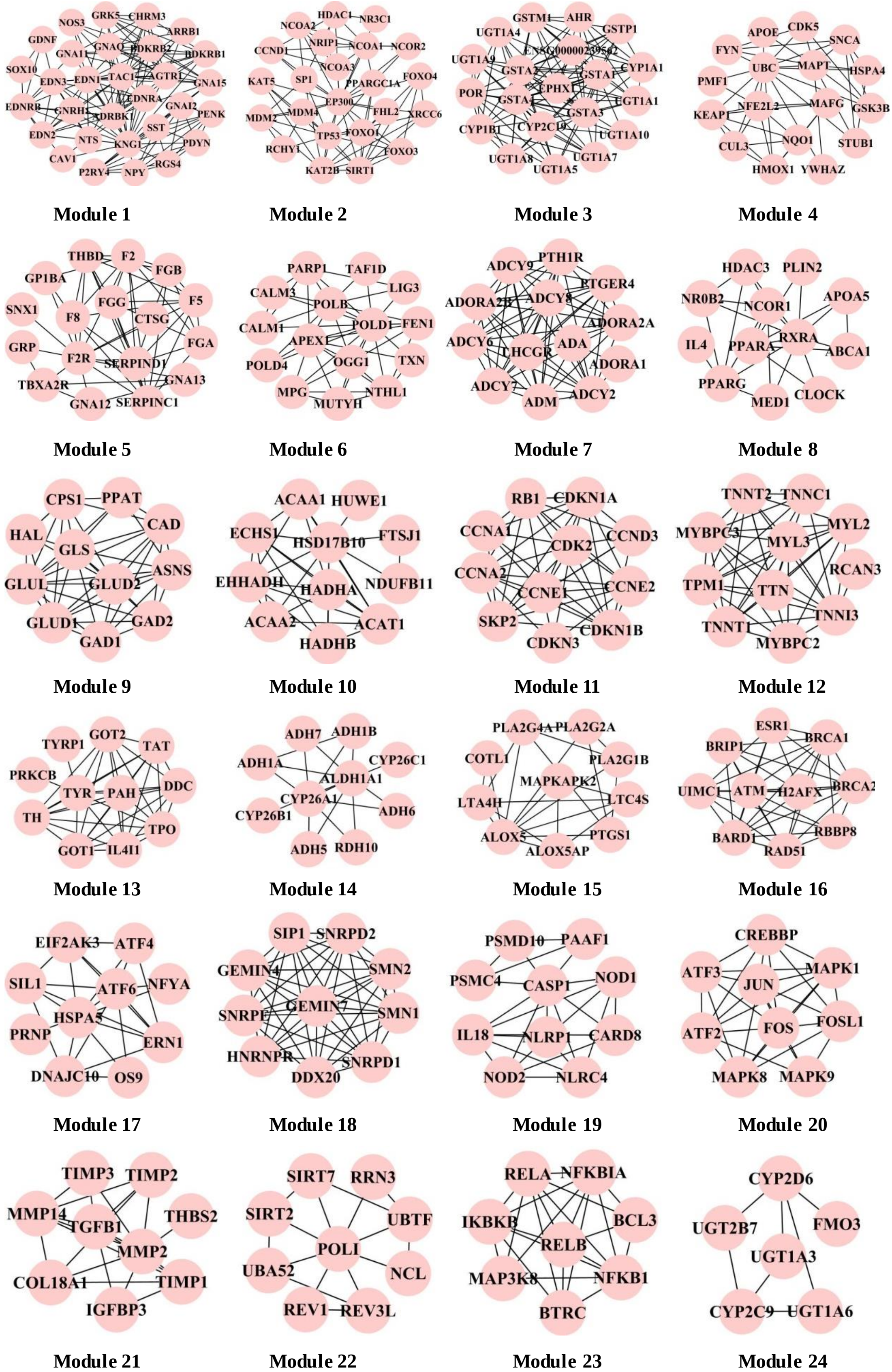
GRK5 CHRM3 NOS3 ARRB1 GDNF GNAQ BDKRB2 BDKRB1 GNA11 SOX10 EDN3 EDN1 TAC1 AGTR1 GNA15 EDNRA EDNRB GNRH1 GNAI2 PENK ADRBK1 SST EDN2 NTS KNG1 PDYN CAV1 RGS4 P2RY4 NPY
Module 1
HDAC1 NR3C1 NCOA2 NRIP1 NCOA1 NCOR2 CCND1 NCOA3 FOXO4 KAT5 SP1 PPARGC1A EP300 MDM2 MDM4 FHL2 XRCC6 TP53 FOXO1 RCHY1 FOXO3 KAT2B SIRT1
Module 2
GSTM1 AHR GSTP1 UGT1A4 ENSG00000239562 UGT1A9 CYP1A1 GSTA2 GSTA1 EPHX1 POR GSTA4 UGT1A1 GSTA3 CYP1B1 CYP2C19 UGT1A10 UGT1A7 UGT1A8 UGT1A5
Module 3
APOE CDK5 SNCA FYN UBC MAPT HSPA4 PMF1 MAFG GSK3B NFE2L2 KEAP1 NQO1 CUL3 STUB1 HMOX1 YWHAZ
Module 4
THBD F2 FGB GP1BA FGG F5 SNX1 F8 CTSG FGA GRP F2R SERPIND1 GNA13 TBXA2R GNA12 SERPINC1
Module 5
PARP1 TAF1D LIG3 CALM3 POLB CALM1 POLD1 FEN1 APEX1 POLD4 OGG1 TXN MPG NTHL1 MUTYH
Module 6
ADCY9 PTH1R PTGER4 ADORA2B ADCY8 ADORA2A ADCY6 ADA THCGR ADORA1 ADCY7 ADCY2 ADM
Module 7
HDAC3 PLIN2 APOA5 NR0B2 NCOR1 RXRA IL4 PPARA ABCA1 PPARG MED1 CLOCK
Module 8
CPS1 PPAT CAD HAL GLS ASNS GLUL GLUD2 GLUD1 GAD2 GAD1
Module 9
ACAA1 HUWE1 ECHS1 HSD17B10 FTSJ1 EHHADH HADHA NDUFB11 ACAA2 ACAT1 HADHB
Module 10
RB1 CDKN1A CCNA1 CDK2 CCND3 CCNA2 CCNE1 CCNE2 SKP2 CDKN1B CDKN3
Module 11
TNNT2 TNNC1 MYBPC3 MYL3 MYL2 RCAN3 TPM1 TTN TNNI3 TNNT1 MYBPC2
Module 12
GOT2 TYRP1 TAT PRKCB TYR PAH DDC TH TPO GOT1 IL4I1
Module 13
ADH7 ADH1B ADH1A CYP26C1 ALDH1A1 CYP26A1 ADH6 CYP26B1 ADH5 RDH10
Module 14
PLA2G4A PLA2G2A COTL1 PLA2G1B MAPKAPK2 LTA4H LTC4S ALOX5 PTGS1 ALOX5AP
Module 15
ESR1 BRIP1 BRCA1 UIMC1 ATM H2AFX BRCA2 BARD1 RBBP8 RAD51
Module 16
EIF2AK3 ATF4 SIL1 ATF6 NFYA HSPA5 PRNP ERN1 DNAJC10 OS9
Module 17
SIP1 SNRPD2 GEMIN4 SMN2 GEMIN7 SNRPE SMN1 HNRNPR SNRPD1 DDX20
Module 18
PSMD10 PAAF1 PSMC4 CASP1 NOD1 IL18 NLRP1 CARD8 NOD2 NLRC4
Module 19
CREBBP ATF3 JUN MAPK1 ATF2 FOS FOSL1 MAPK8 MAPK9
Module 20
TIMP3 TIMP2 MMP14 TGFB1 THBS2 MMP2 COL18A1 TIMP1 IGFBP3
Module 21
SIRT7 RRN3 SIRT2 UBTF POLI UBA52 NCL REV1 REV3L
Module 22
RELA NFKBIA IKBKB BCL3 RELB MAP3K8 NFKB1 BTRC
Module 23
CYP2D6 UGT2B7 FMO3 UGT1A3 CYP2C9 UGT1A6
Module 24

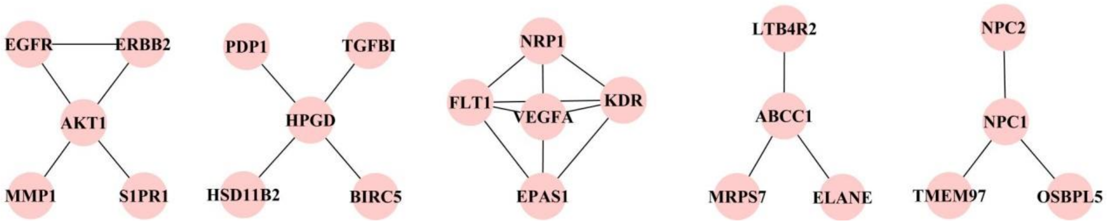

Module 25 **Module 26** **Module 27** **Module 28** **Module 29**

FIGURE S4: Modules in the PIN of *astragalus membranaceus*. With the MCL algorithm, 29 modules are extracted from the network.
