# Supplementary material for: The Mechanism Research of Qishen Yiqi Formula by Module-Network Analysis
Source: Evid Based Complement Alternat Med. 2015 Aug 24;2015:497314. doi: 10.1155/2015/497314 (PMC4561322; doi:10.1155/2015/497314)
Supplement: Supplementary file 1 — Table S1 is about the additional targets' information of each active components from QSYQ. Tables S2–S5 are respective about the results of functional enrichment analysis of salvia miltiorrhiza, Panax notoginseng, Dalbergia odorifera,and Astragalus membranaceus. Figures S1–S4 are respective about the Modules in the PIN of salvia miltiorrhiza, panax notoginseng, dalbergia odorifera, astragalus membranaceus. [file 497314.f1.zip › supplementary materials/TABLE S1 The additional targets' information of the active components of DQAFs.pdf]

Table S1: The additional targets' information of the active components of QSYQ

Active ingredients	Targets	UniProt ID	Source
tanshinone IIA	AKR1B1	P15121	ChEMBL
tanshinone IIA	ALDH1A1	P00352	ChEMBL
tanshinone IIA	ALOX12	P18054	ChEMBL
tanshinone IIA	APAF1	O14727	ChEMBL
tanshinone IIA	ATAD5	Q96QE3	ChEMBL
tanshinone IIA	BAZ2B	Q9UIF8	ChEMBL
tanshinone IIA	CBFB	Q13951	ChEMBL
tanshinone IIA	CBX1	Q6IBN6	ChEMBL
tanshinone IIA	EHMT2	Q96KQ7	ChEMBL
tanshinone IIA	FEN1	P39748	ChEMBL
tanshinone IIA	HPGD	P15428	ChEMBL
tanshinone IIA	HSD17B10	Q99714	ChEMBL
tanshinone IIA	KAT2A	Q92830	ChEMBL
tanshinone IIA	KDM4A	O75164	ChEMBL
tanshinone IIA	KDM4DL	B2RXH2	ChEMBL
tanshinone IIA	L3MBTL1	Q9Y468	ChEMBL
tanshinone IIA	LMNA	P02545	ChEMBL
tanshinone IIA	MAPK1	P63085	ChEMBL
tanshinone IIA	MAPT	P10636	ChEMBL
tanshinone IIA	MBNL1	Q86VM6	ChEMBL
tanshinone IIA	MEN1	O00255	ChEMBL
tanshinone IIA	MLL	Q03164	ChEMBL
tanshinone IIA	NFE2L2	Q68FB5	ChEMBL
tanshinone IIA	POLB	P06746	ChEMBL
tanshinone IIA	POLH	Q9Y253	ChEMBL
tanshinone IIA	POLI	Q9UNA4	ChEMBL
tanshinone IIA	POLK	Q9UBT6	ChEMBL
tanshinone IIA	RECQL	P46063	ChEMBL
tanshinone IIA	RGS4	P49798	ChEMBL
tanshinone IIA	RUNX1	Q01196	ChEMBL
tanshinone IIA	SMAD3	P84022	ChEMBL
tanshinone IIA	TDP1	Q9NUW8	ChEMBL
tanshinone IIA	TP53	P04637	ChEMBL
tanshinone IIA	USP1	O94782	ChEMBL
tanshinone IIA	VDR	P11473	ChEMBL
tanshinone IIA	CD40	P25942	STITCH
tanshinone IIA	CYP1A1	P04798	STITCH
tanshinone IIA	CYP3A4	Q6GRK0	STITCH
tanshinone IIA	FSD1	Q9BTV5	STITCH
tanshinone IIA	HMGB1	P09429	STITCH
tanshinone IIA	KCNE1	Q6FHJ6	STITCH
tanshinone IIA	NFKB1	P19838	STITCH

tanshinone IIA	SPG7	Q9UQ90	STITCH
tanshinone IIA	TNF	P01375	STITCH
tanshinone IIA	PTAFR	P25105	Pharmacophore
cryptotanshinone	ALDH1A1	P00352	ChEMBL
cryptotanshinone	ATAD5	Q96QE3	ChEMBL
cryptotanshinone	BAZ2B	Q9UIF8	ChEMBL
cryptotanshinone	CBX1	Q6IBN6	ChEMBL
cryptotanshinone	FEN1	P39748	ChEMBL
cryptotanshinone	GLP1R	P43220	ChEMBL
cryptotanshinone	GMNN	O75496	ChEMBL
cryptotanshinone	IDH1	O75874	ChEMBL
cryptotanshinone	KAT2A	Q92830	ChEMBL
cryptotanshinone	KDM4A	O75164	ChEMBL
cryptotanshinone	KDM4E	B2RXH2	ChEMBL
cryptotanshinone	RGS4	P49798	ChEMBL
cryptotanshinone	L3MBTL1	Q9Y468	ChEMBL
cryptotanshinone	MAPT	P10636	ChEMBL
cryptotanshinone	MBNL1	Q86VM6	ChEMBL
cryptotanshinone	MLL	Q03164	ChEMBL
cryptotanshinone	NFE2L2	Q68FB5	ChEMBL
cryptotanshinone	NPSR1	B7ZMA2	ChEMBL
cryptotanshinone	POLB	P06746	ChEMBL
cryptotanshinone	POLH	Q9Y253	ChEMBL
cryptotanshinone	POLI	Q9UNA4	ChEMBL
cryptotanshinone	POLK	Q9UBT6	ChEMBL
cryptotanshinone	RECQL	P46063	ChEMBL
cryptotanshinone	RGS4	P49798	ChEMBL
cryptotanshinone	TDP1	Q9NUW8	ChEMBL
cryptotanshinone	TERT	O14746	ChEMBL
cryptotanshinone	THRB	P10828	ChEMBL
cryptotanshinone	VDR	P11473	ChEMBL
cryptotanshinone	WRN	Q14191	ChEMBL
cryptotanshinone	BCL2	P10415	STITCH
cryptotanshinone	CYP3A4	Q6GRK0	STITCH
cryptotanshinone	EDN1	Q6FH53	STITCH
cryptotanshinone	PTGS2	P35354	STITCH
cryptotanshinone	STAT3	P40763	STITCH
salvianolic acid A	LCK	P06239	ChEMBL
salvianolic acid A	SRC	P12931	ChEMBL
salvianolic acid A	STAT3	P40763	ChEMBL
salvianolic acid A	STAT5B	P51692	ChEMBL
salvianolic acid A	STAT1	P42224	ChEMBL
salvianolic acid A	GPT2	Q8TD30	STITCH
salvianolic acid A	GPT	P24298	STITCH

salvianolic acid A	ACE	P12821	Pharmacophore
salvianolic acid A	ADORA2B	P29275	Pharmacophore
salvianolic acid A	AGTR1	Q53YY0	Pharmacophore
salvianolic acid A	ALOX5	P09917	Pharmacophore
salvianolic acid A	CDK2	P24941	Pharmacophore
salvianolic acid A	EDNRA	P25101	Pharmacophore
salvianolic acid A	EDNRB	P24530	Pharmacophore
salvianolic acid A	F10	P00742	Pharmacophore
salvianolic acid A	F2R	P25116	Pharmacophore
salvianolic acid A	PDE4A	P27815	Pharmacophore
salvianolic acid B	LCK	P06239	ChEMBL
salvianolic acid B	SRC	P12931	ChEMBL
salvianolic acid B	STAT1	P42224	ChEMBL
salvianolic acid B	STAT3	P40763	ChEMBL
salvianolic acid B	STAT5B	P51692	ChEMBL
salvianolic acid B	BAX	Q07812	STITCH
salvianolic acid B	BCL2L1	Q07817	STITCH
salvianolic acid B	MMP2	P08253	STITCH
salvianolic acid B	MMP9	P14780	STITCH
salvianolic acid B	PTGS2	P35354	STITCH
salvianolic acid B	ACE	P12821	Pharmacophore
salvianolic acid B	ADORA1	P30542	Pharmacophore
salvianolic acid B	ADORA2B	P29275	Pharmacophore
salvianolic acid B	AGTR1	Q53YY0	Pharmacophore
salvianolic acid B	ALOX5	P09917	Pharmacophore
salvianolic acid B	CDK2	P24941	Pharmacophore
salvianolic acid B	ECE1	P42892	Pharmacophore
salvianolic acid B	EDNRA	P25101	Pharmacophore
salvianolic acid B	EDNRB	P24530	Pharmacophore
salvianolic acid B	F2R	P25116	Pharmacophore
salvianolic acid B	PDE4A	P27815	Pharmacophore
tanshinol	AGTR1	Q53YY0	Pharmacophore
tanshinol	EDNRA	P25101	Pharmacophore
tanshinol	EDNRB	P24530	Pharmacophore
tanshinol	CDK2	P24941	Pharmacophore
tanshinol	ACE	P12821	Pharmacophore
tanshinol	ALOX5	P09917	Pharmacophore
tanshinol	CASP1	P29466	Pharmacophore
tanshinol	F10	P00742	Pharmacophore
protocatechuic aldehyde	POLI	Q9UNA4	ChEMBL
protocatechuic aldehyde	KDM4A	O75164	ChEMBL
protocatechuic aldehyde	GLS	O94925	ChEMBL
protocatechuic aldehyde	KDM4E	B2RXH2	ChEMBL
protocatechuic aldehyde	BAZ2B	Q9UIF8	ChEMBL

protocatechuic aldehyde	EHMT2	Q96KQ7	ChEMBL
protocatechuic aldehyde	RAB9A	P51151	ChEMBL
protocatechuic aldehyde	TNF	P01375	STITCH
protocatechuic aldehyde	PLA2G7	Q13093	Pharmacophore
dencichine	GLRX	P35754	STITCH
dencichine	GRIK1	P39086	STITCH
dencichine	CYP3A4	Q6GRK0	Pharmacophore
dencichine	AKR1C3	P42330	Pharmacophore
dencichine	PDE5A	O76074	Pharmacophore
dencichine	PDE4B	Q07343	Pharmacophore
dencichine	ACOT8	O14734	Pharmacophore
ginsenoside Rb1	TOP1	P11387	ChEMBL
ginsenoside Rb1	TOP2A	P11388	ChEMBL
ginsenoside Rb1	ESR1	P03372	STITCH
ginsenoside Rb1	FOS	P01100	STITCH
ginsenoside Rb1	PGR	Q5NUL3	STITCH
ginsenoside Rb1	PIK3R1	P27986	STITCH
ginsenoside Rb1	PTGS2	P35354	STITCH
ginsenoside Rb1	RB1	P06400	STITCH
ginsenoside Rb1	RBL2	Q08999	STITCH
ginsenoside Rb1	RGN	Q15493	STITCH
ginsenoside Rb1	AGTR1	Q53YY0	Pharmacophore
ginsenoside Rb1	EDNRB	P24530	Pharmacophore
ginsenoside Rb1	PDE4A	P27815	Pharmacophore
ginsenoside Rb1	ADORA1	P30542	Pharmacophore
ginsenoside Rb1	ADORA2B	P29275	Pharmacophore
ginsenoside Rg1	IL2	P60568	STITCH
ginsenoside Rg1	NPM1	P06748	STITCH
ginsenoside Rg1	NR3C1	P04150	STITCH
ginsenoside Rg1	RB1	P06400	STITCH
ginsenoside Rg1	RBL2	Q08999	STITCH
ginsenoside Rg1	STMN4	Q9H169	STITCH
ginsenoside Rg1	THBS1	A0PJG0	STITCH
ginsenoside Rg1	TNF	P01375	STITCH
ginsenoside Rg1	VEGFA	P15692	STITCH
ginsenoside Rg1	ADORA2B	P29275	Pharmacophore
ginsenoside Rg1	ADORA1	P30542	Pharmacophore
ginsenoside Rg1	AGTR1	Q53YY0	Pharmacophore
ginsenoside Rg1	CASP1	P29466	Pharmacophore
ginsenoside Rg1	EDNRA	P25101	Pharmacophore
ginsenoside Rg1	EDNRB	P24530	Pharmacophore
ginsenoside Rg1	F2R	P25116	Pharmacophore
ginsenoside Rg1	HMGCR	P04035	Pharmacophore
ginsenoside Rg1	PDE4A	P27815	Pharmacophore

notoginsenoside R1	RB1	P06400	STITCH
notoginsenoside R1	RBL2	Q08999	STITCH
notoginsenoside R1	RGN	Q15493	STITCH
notoginsenoside R1	STMN4	Q9H169	STITCH
notoginsenoside R1	TNF	P01375	STITCH
notoginsenoside R1	ADORA1	P30542	Pharmacophore
notoginsenoside R1	ADORA2B	P29275	Pharmacophore
notoginsenoside R1	AGTR1	Q53YY0	Pharmacophore
notoginsenoside R1	CASP1	P29466	Pharmacophore
notoginsenoside R1	EDNRB	P24530	Pharmacophore
notoginsenoside R1	PDE4A	P27815	Pharmacophore
butein	EGFR	Q504U8	ChEMBL
butein	FGFR1	P11362	ChEMBL
butein	FGFR2	P21802	ChEMBL
butein	GBA	P04062	ChEMBL
butein	HSPA5	P11021	ChEMBL
butein	KDR	P35968	ChEMBL
butein	APEX1	Q5TZP7	ChEMBL
butein	CDK2	P24941	ChEMBL
butein	KIT	P10721	ChEMBL
butein	MET	P08581	ChEMBL
butein	POLK	Q9UBT6	ChEMBL
butein	SRC	P12931	ChEMBL
butein	VDR	P11473	ChEMBL
butein	BCL2	P10415	STITCH
butein	CXCR4	P61073	STITCH
butein	MMP7	P09237	STITCH
butein	MMP9	P14780	STITCH
butein	PTPN3	P26045	STITCH
butein	PTPN6	P29350	STITCH
butein	STAT3	P40763	STITCH
butein	TNF	P01375	STITCH
butein	TNFRSF10B	Q6FH58	STITCH
butein	TNFSF10	Q6IBA9	STITCH
formononetin	AL1A1	P00352	ChEMBL
formononetin	NF2L2	Q16236	ChEMBL
formononetin	MAPT	P10636	ChEMBL
formononetin	KDM4E	B2RXH2	ChEMBL
formononetin	SMN1	Q16637	ChEMBL
formononetin	HPGD	P15428	ChEMBL
formononetin	HSPA5	P11021	ChEMBL
formononetin	GLS	O94925	ChEMBL
formononetin	CP1A1	P04798	ChEMBL
formononetin	PPARG	P37231	ChEMBL

formononetin	PPARA	Q07869	ChEMBL
formononetin	CP2D6	P10635	ChEMBL
formononetin	ABCC1	P33527	ChEMBL
formononetin	CP2C9	P11712	ChEMBL
formononetin	CP3A4	P08684	ChEMBL
formononetin	CP2CJ	P33261	ChEMBL
formononetin	SLC5A2	P31639	ChEMBL
formononetin	TP53	P04637	ChEMBL
formononetin	HSD17B10	Q99714	ChEMBL
formononetin	SLC5A1	A4QPH0	ChEMBL
formononetin	BRCA1	Q3B891	ChEMBL
formononetin	CASP1	P29466	Pharmacophore
formononetin	BDKRB2	P30411	Pharmacophore
formononetin	ALOX5	P09917	Pharmacophore
formononetin	EDNRB	P24530	Pharmacophore
formononetin	CDK2	P24941	Pharmacophore
formononetin	AGTR1	Q53YY0	Pharmacophore
isoliquiritigenin	AKR1B1	P15121	ChEMBL
isoliquiritigenin	NFE2L2	Q68FB5	ChEMBL
isoliquiritigenin	MAPT	P10636	ChEMBL
isoliquiritigenin	LMNA	P02545	ChEMBL
isoliquiritigenin	BLM	P54132	ChEMBL
isoliquiritigenin	HPGD	P15428	ChEMBL
isoliquiritigenin	SMAD3	P84022	ChEMBL
isoliquiritigenin	GMNN	O75496	ChEMBL
isoliquiritigenin	PMP22	Q01453	ChEMBL
isoliquiritigenin	MLL	Q03164	ChEMBL
isoliquiritigenin	CBX1	Q6IBN6	ChEMBL
isoliquiritigenin	ATAD5	Q96QE3	ChEMBL
isoliquiritigenin	MPHOSPH8	Q99549	ChEMBL
isoliquiritigenin	HSPA5	P11021	ChEMBL
isoliquiritigenin	GLP1R	P43220	ChEMBL
isoliquiritigenin	ATXN2	Q24JQ7	ChEMBL
isoliquiritigenin	USP1	O94782	ChEMBL
isoliquiritigenin	KIT	P10721	ChEMBL
isoliquiritigenin	VDR	P11473	ChEMBL
isoliquiritigenin	EGFR	Q504U8	ChEMBL
isoliquiritigenin	DRD1	P21728	ChEMBL
isoliquiritigenin	GBA	P04062	ChEMBL
isoliquiritigenin	PIN1	Q13526	ChEMBL
isoliquiritigenin	PPARG	P37231	ChEMBL
isoliquiritigenin	KCNH2	Q12809	ChEMBL
isoliquiritigenin	SRC	P12931	ChEMBL
isoliquiritigenin	KDR	P35968	ChEMBL

isoliquiritigenin	MTOR	P42345	ChEMBL
isoliquiritigenin	MMP9	P14780	ChEMBL
isoliquiritigenin	PTPN1	P18031	ChEMBL
isoliquiritigenin	CYP2C9	P11712	ChEMBL
isoliquiritigenin	CYP3A4	Q6GRK0	ChEMBL
isoliquiritigenin	CYP2C19	P33261	ChEMBL
isoliquiritigenin	FGFR1	P11362	ChEMBL
isoliquiritigenin	MET	P08581	ChEMBL
isoliquiritigenin	TP53	P04637	ChEMBL
isoliquiritigenin	FGFR2	P21802	ChEMBL
isoliquiritigenin	JUN	P05412	ChEMBL
isoliquiritigenin	FEN1	P39748	ChEMBL
isoliquiritigenin	NPSR1	B7ZMA2	ChEMBL
isoliquiritigenin	POLK	Q9UBT6	ChEMBL
isoliquiritigenin	POLI	Q9UNA4	ChEMBL
isoliquiritigenin	RELA	Q2TAM5	ChEMBL
isoliquiritigenin	APEX1	Q5TZP7	ChEMBL
isoliquiritigenin	EHMT2	Q96KQ7	ChEMBL
isoliquiritigenin	ALOX12	P18054	STITCH
isoliquiritigenin	ALOX12B	O75342	STITCH
isoliquiritigenin	CASP3	P42574	STITCH
isoliquiritigenin	NFKBIA	P25963	STITCH
isoliquiritigenin	SLC2A1	P11166	STITCH
isoliquiritigenin	TNF	P01375	STITCH
nerolidol	ALOX15	P16050	ChEMBL
nerolidol	THRB	P10828	ChEMBL
nerolidol	USP1	O94782	ChEMBL
calycosin	APEX1	Q5TZP7	ChEMBL
calycosin	BRCA1	Q3B891	ChEMBL
calycosin	KDM4E	B2RXH2	ChEMBL
calycosin	MAPT	P10636	ChEMBL
calycosin	NPC1	O15118	ChEMBL
calycosin	POLB	P06746	ChEMBL
calycosin	POLI	Q9UNA4	ChEMBL
calycosin	RAB9A	P51151	ChEMBL
astragaloside I	NFKB1	P19838	STITCH
astragaloside I	ADORA1	P30542	Pharmacophore
astragaloside I	ADORA2B	P29275	Pharmacophore
astragaloside I	AGTR1	Q53YY0	Pharmacophore
astragaloside I	EDNRA	P25101	Pharmacophore
astragaloside I	EDNRB	P24530	Pharmacophore
astragaloside I	F2R	P25116	Pharmacophore
astragaloside IV	TYR	P14679	ChEMBL
astragaloside IV	MMP2	P08253	STITCH

astragaloside IV	TNNI3	P19429	STITCH
astragaloside IV	ADORA1	P30542	Pharmacophore
astragaloside IV	ADORA2B	P29275	Pharmacophore
astragaloside IV	AGTR1	Q53YY0	Pharmacophore
astragaloside IV	CASP1	P29466	Pharmacophore
astragaloside IV	EDNRA	P25101	Pharmacophore
astragaloside IV	EDNRB	P24530	Pharmacophore
astragaloside IV	F2R	P25116	Pharmacophore
