# Supplementary material for: The Mechanism Research of Qishen Yiqi Formula by Module-Network Analysis
Source: Evid Based Complement Alternat Med. 2015 Aug 24;2015:497314. doi: 10.1155/2015/497314 (PMC4561322; doi:10.1155/2015/497314)
Supplement: Supplementary file 1 — Table S1 is about the additional targets' information of each active components from QSYQ. Tables S2–S5 are respective about the results of functional enrichment analysis of salvia miltiorrhiza, Panax notoginseng, Dalbergia odorifera,and Astragalus membranaceus. Figures S1–S4 are respective about the Modules in the PIN of salvia miltiorrhiza, panax notoginseng, dalbergia odorifera, astragalus membranaceus. [file 497314.f1.zip › supplementary materials/TABLE S2 GO biological process terms of the modules of salvia miltiorrhiza.pdf]

TABLE S2: GO biological process terms of the modules of *salvia miltiorrhiza*

Modules	p-value	GO terms
1	6.20E-23	regulation of cAMP metabolic process
2	6.26E-33	DNA repair
3	4.37E-16	G-protein coupled receptor signaling pathway
4	1.74E-11	DNA repair
5	9.10E-22	lipid metabolic process
6	5.94E-13	inflammatory response
7	9.19E-20	tricarboxylic acid cycle
8	6.81E-17	apoptotic signaling pathway
9	2.44E-15	RNA biosynthetic process
10	1.24E-11	interferon-gamma-mediated signaling pathway
11	1.13E-16	apoptotic process
12	2.83E-16	chromatin organization
13	2.33E-17	immune response-regulating signaling pathway
14	3.77E-08	lipid catabolic process
15	1.19E-22	cell cycle phase transition
16	2.71E-06	regulation of RNA metabolic process
17	1.61E-20	xenobiotic metabolic process
18	2.53E-06	regulation of RNA splicing
19	1.13E-09	carbohydrate metabolic process
20	8.28E-16	G-protein coupled receptor signaling pathway
21	1.25E-19	cell cycle phase transition
22	3.71E-08	regulation of blood coagulation
23	1.39E-15	potassium ion transport
24	2.31E-06	execution phase of apoptosis
25	7.03E-07	regulation of apoptotic process
26	3.93E-07	positive regulation of cellular protein metabolic process
27	6.72E-10	regulation of immune response
28	3.37E-18	xenobiotic metabolic process
29	5.27E-11	DNA-dependent transcription
30	5.23E-08	regulation of immune response
31	3.74E-11	regulation of interleukin-1 secretion
32	3.18E-06	negative regulation of RNA metabolic process
33	8.15E-09	positive regulation of RNA metabolic process
34	4.39E-13	xenobiotic metabolic process
35	4.50E-07	DNA metabolic process
36	9.06E-11	negative regulation of cAMP biosynthetic process
37	1.68E-09	toll-like receptor signaling pathway
38	1.32E-05	negative regulation of protein metabolic process
39	4.99E-08	RNA biosynthetic process
40	8.42E-10	toll-like receptor signaling pathway
41	1.11E-07	DNA metabolic process
42	7.37E-06	positive regulation of RNA metabolic process

43	1.97E-05	cAMP metabolic process
44	1.11E-05	DNA packaging
45	2.04E-04	chromatin organization
46	1.21E-06	defense response
47	9.70E-06	transcription from RNA polymerase I promoter
48	7.04E-07	regulation of blood coagulation
49	6.11E-06	RNA biosynthetic process
50	1.11E-07	toll-like receptor signaling pathway
51	1.31E-04	lipoxygenase pathway
52	6.90E-04	alanine catabolic process
53	1.27E-11	neural crest cell migration
54	2.18E-06	apoptotic process
55	6.76E-05	RNA biosynthetic process
56	4.99E-04	DNA metabolic process
57	1.67E-06	platelet activation

Notes: P-value is the probability of obtaining the observed effect, a very small P-value indicates that the observed effect is very unlikely to have arisen purely by chance, and therefore provides evidence against the null hypothesis.
