# Supplementary material for: The Mechanism Research of Qishen Yiqi Formula by Module-Network Analysis
Source: Evid Based Complement Alternat Med. 2015 Aug 24;2015:497314. doi: 10.1155/2015/497314 (PMC4561322; doi:10.1155/2015/497314)
Supplement: Supplementary file 1 — Table S1 is about the additional targets' information of each active components from QSYQ. Tables S2–S5 are respective about the results of functional enrichment analysis of salvia miltiorrhiza, Panax notoginseng, Dalbergia odorifera,and Astragalus membranaceus. Figures S1–S4 are respective about the Modules in the PIN of salvia miltiorrhiza, panax notoginseng, dalbergia odorifera, astragalus membranaceus. [file 497314.f1.zip › supplementary materials/TABLE S3 GO biological process terms of the modules of panax notoginseng.pdf]

Table S3: GO biological process terms of the modules of *panax notoginseng*

Modules	p-value	GO terms
1	2.888E-21	G-protein coupled receptor signaling pathway
2	2.363E-16	cell cycle
3	4.133E-16	cell cycle
4	1.203E-15	nucleotide metabolic process
5	1.461E-16	G-protein coupled receptor signaling pathway
6	9.556E-23	xenobiotic metabolic process
7	2.42E-14	lipid metabolic process
8	1.291E-12	toll-like receptor signaling pathway
9	1.072E-13	transmembrane receptor protein tyrosine kinase signaling pathway
10	6.229E-11	regulation of interleukin-1 secretion
11	3.727E-10	xenobiotic metabolic process
12	5.738E-11	regulation of blood coagulation
13	1.077E-10	regulation of inflammatory response
14	2.081E-08	negative regulation of inflammatory response
15	6.161E-11	vascular endothelial growth factor receptor signaling pathway
16	4.079E-06	apoptotic process
17	1.355E-07	positive regulation of RNA metabolic process
18	1.326E-09	cell cycle
19	5.579E-09	cellular lipid metabolic process
20	3.841E-08	lipid metabolic process
21	3.239E-08	copper ion import
22	1.406E-05	cAMP metabolic process
23	0.0002926	cellular lipid metabolic process
24	4.625E-07	regulation of blood coagulation
25	2.904E-08	transmission of nerve impulse

Notes: P-value is the probability of obtaining the observed effect, a very small P-value indicates that the observed effect is very unlikely to have arisen purely by chance, and therefore provides evidence against the null hypothesis.
