# Supplementary material for: The Mechanism Research of Qishen Yiqi Formula by Module-Network Analysis
Source: Evid Based Complement Alternat Med. 2015 Aug 24;2015:497314. doi: 10.1155/2015/497314 (PMC4561322; doi:10.1155/2015/497314)
Supplement: Supplementary file 1 — Table S1 is about the additional targets' information of each active components from QSYQ. Tables S2–S5 are respective about the results of functional enrichment analysis of salvia miltiorrhiza, Panax notoginseng, Dalbergia odorifera,and Astragalus membranaceus. Figures S1–S4 are respective about the Modules in the PIN of salvia miltiorrhiza, panax notoginseng, dalbergia odorifera, astragalus membranaceus. [file 497314.f1.zip › supplementary materials/TABLE S4 GO biological process terms of the modules of dalbergia odorifera.pdf]

Table S4: GO biological process terms of the modules of *dalbergia odorifera*

| Modules | p-value | GO terms |
|---|---|---|
| 1 | 3.992E-17 | regulation of cell death |
| 2 | 1.982E-23 | regulation of cAMP metabolic process |
| 3 | 4.068E-32 | DNA repair |
| 4 | 3.64E-24 | transmembrane receptor protein tyrosine kinase signaling pathway |
| 5 | 4.561E-17 | RNA biosynthetic process |
| 6 | 5.011E-16 | DNA damage checkpoint |
| 7 | 4.799E-25 | apoptotic process |
| 8 | 2.203E-33 | xenobiotic metabolic process |
| 9 | 7.861E-15 | G-protein coupled receptor signaling pathway |
| 10 | 5.994E-13 | positive regulation of RNA metabolic process |
| 11 | 4.262E-14 | transmembrane receptor protein tyrosine kinase signaling pathway |
| 12 | 1.189E-22 | cell cycle phase transition |
| 13 | 1.547E-06 | regulation of protein metabolic process |
| 14 | 4.773E-05 | RNA metabolic process |
| 15 | 2.03E-27 | potassium ion transport |
| 16 | 2.706E-06 | regulation of RNA metabolic process |
| 17 | 2.065E-11 | cellular lipid metabolic process |
| 18 | 2.379E-14 | lipid metabolic process |
| 19 | 1.128E-09 | carbohydrate metabolic process |
| 20 | 2.499E-22 | cellular amino acid catabolic process |
| 21 | 3.767E-08 | lipid catabolic process |
| 22 | 3.684E-11 | RNA biosynthetic process |
| 23 | 1.252E-19 | cell cycle phase transition |
| 24 | 1.699E-12 | apoptotic process |
| 25 | 3.268E-12 | xenobiotic metabolic process |
| 26 | 9.255E-19 | mRNA processing |
| 27 | 2.036E-14 | transmembrane receptor protein tyrosine kinase signaling pathway |
| 28 | 2.315E-06 | execution phase of apoptosis |
| 29 | 6.229E-11 | regulation of interleukin-1 secretion |
| 30 | 2.361E-13 | toll-like receptor signaling pathway |
| 31 | 7.594E-06 | inflammatory response |
| 32 | 6.836E-05 | regulation of cell proliferation |
| 33 | 3.177E-06 | negative regulation of RNA metabolic process |
| 34 | 1.672E-18 | insulin receptor signaling pathway |
| 35 | 1.174E-10 | cell cycle phase |
| 36 | 2.65E-07 | immune response-regulating signaling pathway |
| 37 | 1.364E-11 | regulation of inflammatory response |
| 38 | 3.78E-18 | vascular endothelial growth factor receptor signaling pathway |
| 39 | 5.556E-07 | transmission of nerve impulse |
| 40 | 7.042E-07 | regulation of blood coagulation |
| 41 | 8.928E-06 | transcription initiation from RNA polymerase I promoter |
| 42 | 8.087E-12 | insulin receptor signaling pathway |

| | | |
|---|---|---|
| 43 | 3.221E-09 | xenobiotic metabolic process |
| 44 | 4.173E-05 | transcription from RNA polymerase II promoter |
| 45 | 0.0006571 | positive regulation of exit from mitosis |
| 47 | 3.882E-06 | regulation of response to interferon-gamma |
| 49 | 1.273E-11 | neural crest cell migration |

*Notes*: P-value is the probability of obtaining the observed effect, a very small P-value indicates that the observed effect is very unlikely to have arisen purely by chance, and therefore provides evidence against the null hypothesis.
