# Supplementary material for: The Mechanism Research of Qishen Yiqi Formula by Module-Network Analysis
Source: Evid Based Complement Alternat Med. 2015 Aug 24;2015:497314. doi: 10.1155/2015/497314 (PMC4561322; doi:10.1155/2015/497314)
Supplement: Supplementary file 1 — Table S1 is about the additional targets' information of each active components from QSYQ. Tables S2–S5 are respective about the results of functional enrichment analysis of salvia miltiorrhiza, Panax notoginseng, Dalbergia odorifera,and Astragalus membranaceus. Figures S1–S4 are respective about the Modules in the PIN of salvia miltiorrhiza, panax notoginseng, dalbergia odorifera, astragalus membranaceus. [file 497314.f1.zip › supplementary materials/TABLE S5 GO biological process terms of the modules of astragalus membranaceus.pdf]

Table S5: GO biological process terms of the modules of *astragalus membranaceus*

Modules	p-value	GO terms
1	3.30E-19	regulation of blood vessel size
2	1.15E-17	positive regulation of RNA metabolic process
3	7.10E-35	xenobiotic metabolic process
4	2.68E-10	regulation of apoptotic process
5	1.17E-22	blood coagulation
6	5.23E-12	DNA metabolic process
7	1.27E-13	cyclic nucleotide metabolic process
8	7.98E-11	cellular lipid metabolic process
9	2.50E-22	cellular amino acid catabolic process
10	8.04E-12	fatty acid beta-oxidation
11	1.25E-19	cell cycle phase transition
12	2.77E-29	muscle filament sliding
13	1.57E-11	cellular amino acid metabolic process
14	1.68E-17	xenobiotic metabolic process
15	4.10E-10	cellular lipid metabolic process
16	1.83E-11	DNA metabolic process
17	1.03E-11	positive regulation of nuclease activity
18	3.52E-17	mRNA metabolic process
19	2.74E-13	positive regulation of interleukin-1 beta production
20	9.09E-15	toll-like receptor 1 signaling pathway
21	2.95E-07	regulation of cell migration
22	1.38E-06	cellular macromolecule biosynthetic process
23	3.48E-09	positive regulation of cytokine production
24	1.92E-11	xenobiotic metabolic process
25	9.07E-07	epidermal growth factor receptor signaling pathway
26	1.64E-04	lipoxygenase pathway
27	2.70E-10	angiogenesis
29	2.91E-08	cholesterol transport

Notes: P-value is the probability of obtaining the observed effect, a very small P-value indicates that the observed effect is very unlikely to have arisen purely by chance, and therefore provides evidence against the null hypothesis.
